# Microbial Degradation of Free and Halogenated Estrogens in River Water-Sediment Microcosms

**DOI:** 10.1021/acs.est.3c00801

**Published:** 2023-07-10

**Authors:** David R. Griffith, MacKayla Carolan, Manuel Marcos Gutierrez, Anya Romig, Nathan Garcia-Diaz, Carolyn P. Hutchinson, Rosa León Zayas

**Affiliations:** Willamette University, 900 State Street, Salem, Oregon 97301, United States

**Keywords:** estrogen, halogenated estrogen, biodegradation, kinetics, sorption, oxidation, transformation product, river water

## Abstract

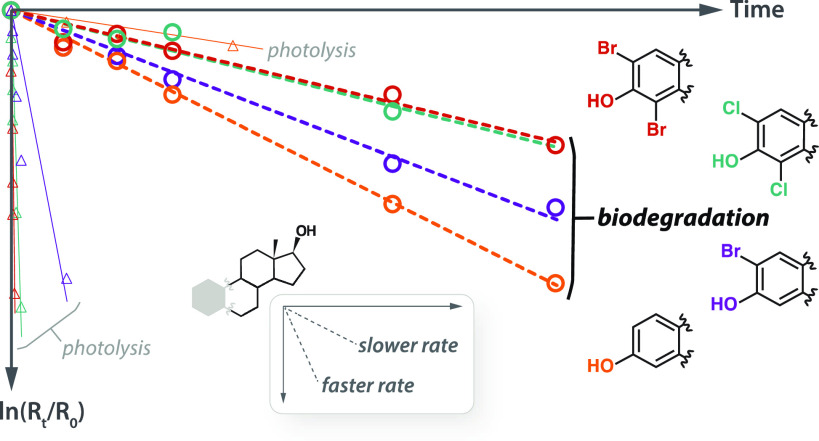

Halogenated estrogens are formed during chlorine-based wastewater disinfection and have been detected in wastewater treatment plant effluent; however, very little is known about their susceptibility to biodegradation in natural waters. To better understand the biodegradation of free and halogenated estrogens in a large river under environmentally relevant conditions, we measured estrogen kinetics in aerobic microcosms containing water and sediment from the Willamette River (OR, USA) at two concentrations (50 and 1250 ng L^–1^). Control microcosms were used to characterize losses due to sorption and other abiotic processes, and microbial dynamics were monitored using 16S rRNA gene sequencing and ATP. We found that estrogen biodegradation occurred on timescales of hours to days and that in river water spiked at 50 ng L^–1^ half-lives were significantly shorter for 17β-estradiol (*t*_1/2,bio_ = 42 ± 3 h) compared to its monobromo (*t*_1/2,bio_ = 49 ± 5 h), dibromo (*t*_1/2,bio_ = 88 ± 12 h), and dichloro (*t*_1/2,bio_ = 98 ± 16 h) forms. Biodegradation was also faster in microcosms with high initial estrogen concentrations as well as those containing sediment. Free and halogenated estrone were important transformation products in both abiotic and biotic microcosms. Taken together, our findings suggest that biodegradation is a key process for removing free estrogens from surface waters but likely plays a much smaller role for the more highly photolabile halogenated forms.

## Abstract 2

Little is known about the environmental fate of halogenated estrogens. Our results suggest that these contaminants are removed from natural waters much faster by sunlight exposure than by biodegradation, which has implications for wastewater treatment technologies and mitigating risks to aquatic systems.

## Introduction

Steroidal estrogens are widespread in natural and engineered aquatic systems and can cause endocrine disruption in vertebrates at low part-per-trillion concentrations.^1−3^ Characterizing the sources and transformations of these compounds is the key to understanding their environmental fate. Accurately quantifying removal rates and identifying transformation products under environmentally relevant conditions is of particular value for predicting estrogen concentrations in natural waters and designing effective mitigation strategies.

Rivers often receive large inputs of estrogens via wastewater that is discharged from municipal and/or animal feeding operations.^4,5^ Local wildlife populations may also be significant sources of estrogens for aquatic systems.^5,6^ The processes responsible for estrogen removal from rivers include flushing, sorption, oxidation–reduction reactions, photolysis, and biodegradation.^7−12^ Previous work has focused on the fate of free estrogens and, to a lesser extent, their conjugated forms. These studies have measured concentrations in a range of systems, characterized partition coefficients (e.g., *K*_d_ and *K*_oc_), described reactions catalyzed by metals, investigated photolysis in the presence of standard humic substances, quantified biodegradation rates, and identified likely transformation products.

Halogenated estrogens have received much less attention. These estrogens are formed during chlorine-based disinfection and may represent a large fraction of the total estrogen load in treated municipal wastewater effluent,^13,14^ yet we know very little about the processes controlling their environmental fate. Based on equilibrium partition coefficients (Table S1), halogenated estrogens are expected to sorb more strongly than free estrogens to sediments and soils.^15^ Recent work has also shown that halogenated estrogen photolysis occurs on sub-hour timescales at circumneutral pH, significantly faster than the day–week timescales of free estrogen photolysis under similar conditions.^16^ In the current study, we characterize halogenated estrogen biodegradation, a previously unstudied but potentially important removal pathway.

Biodegradation is thought to be a key process for free estrogen removal, especially under aerobic conditions. Microbes are known to degrade estrogens through a variety of processes that include growth-linked and co-metabolic pathways.^17,18^ To date, most estrogen biodegradation studies have focused on the behavior of free estrogens in soil, sludge, and wastewater matrices. Reported free estrogen biodegradation half-lives range from hours to weeks, depending on the source water and experimental conditions.^7,19−37^ Yet, many of these studies used high (μg–mg L^–1^) initial concentrations^7,38^ and largely neglected microbial community dynamics. Several studies also did not take sorption or abiotic oxidation processes into account. Fewer experiments have been conducted at ng L^–1^ levels using whole river water and sediment collected across a range of seasons,^18,25^ and none have investigated halogenated estrogen biodegradation.

Measuring the rate of halogenated estrogen biodegradation relative to that of free forms and comparing this process to others, such as photolysis, is an important step in identifying dominant removal pathways and modeling environmental fate. These efforts will also shed light on the behavior of other phenolic contaminants that are similarly susceptible to halogenation by free chlorine and chloramines present in water treatment systems.

The aim of the present study was to characterize the biodegradation kinetics of free and halogenated estrogens (Figure S1) in river water-sediment microcosms in order to improve estrogen fate models. Microcosms containing whole water and sediment from a large river were spiked with estrogens at two initial concentrations (50 and 1250 ng L^–1^). Time-course samples from biotic microcosms and abiotic controls were analyzed using liquid chromatography tandem mass spectrometry (LC–MS/MS), and biodegradation rate constants were determined via nonlinear regression analysis. Concurrent measurements of transformation product growth and decay as well as microcosm water chemistry, nutrient levels, ATP concentrations, and microbial diversity (16S rRNA) provided context for biodegradation kinetic data.

## Materials and Methods

### Chemicals, Solvents, and Standards

Sodium azide (NaN_3_; 99.5%), 17β-estradiol (E2, 98%), and estrone (E1, 99%) were purchased from Sigma-Aldrich (St. Louis, MO). The halogenated estrogens, 2-bromo-17β-estradiol (monoBrE2), and 2,4-dibromo-17β-estradiol (diBrE2) were acquired from Steraloids (Newport, RI); 2,4-dichloro-17β-estradiol (diClE2) was supplied by Hiroshi Matsufuji (Nihon University; Japan). These particular E2 derivatives, which were previously detected in wastewater effluent,^13,14^ allowed us to study the effect of both halogen identity (Br vs Cl) and halogenation extent (mono-vs di-) on estrogen biodegradation. Deuterated 17β-estradiol (E2-*d4*, 95%) was sourced from Cambridge Isotope Laboratories (Andover, MA). Methanol (MeOH), dichloromethane (DCM), and ethyl acetate (EtOAc) were HPLC grade (Optima) and purchased from Fisher Scientific (Hampton, NH). Ultrapure water was produced using an ELGA purifier (Veolia Water Technologies; Paris, France).

### Source Water and Experimental Design

River water was collected from the Willamette River at a site (45° 00.47′ N and 123° 04.28′ W) located in the center of the channel, ∼100 m upstream of the Willow Lake Water Pollution Control Facility diffuser outfall, at a depth of 0.5 m below the surface (Table S2). The Willamette River drains a 30,000 km^2^ watershed containing a mixture of forested, agricultural, and urban lands and receives treated wastewater inputs from several medium-sized cities along the main stem. Four separate sampling campaigns were conducted (Table S2), each followed by a set of laboratory microcosm biodegradation experiments during May 2018 (“BD-1805”), October 2018 (“BD-1810”), June 2019 (“BD-1906”), and July 2019 (“BD-1907”). At the time of collection, river discharge ranged from 199 to 425 m^3^ s^–1^, and water depth was between 1.2 and 2.4 m. In situ river water temperatures, dissolved oxygen (DO), and pH ranged from 14.7–20.4 °C, 7.3–9.5 mg L^–1^, and 6.5–7.6, respectively.

### Microcosm Setup

Microcosms consisted of wide-mouth amber glass bottles containing recently collected water (2.0 L) and homogenized wet sediment (50 g) from the Willamette River (Table S3). Autoclaved foam plugs prevented microbial contamination from airborne particles and helped maintain aerobic conditions (8.2 ± 1.0 mg DO L^–1^) by allowing gas exchange. Sodium azide (38 mM) was used to inhibit microbial activity in abiotic control microcosms. Prepared microcosms were allowed to equilibrate for 2–6 days before estrogen spikes were introduced to ensure that the water-sediment system reached equilibrium and that microbial activity was fully inhibited in abiotic controls. Microcosms were spiked with 10 μL (0.0005% v/v) of the appropriate methanolic stock solutions containing single estrogens or mixtures of estrogens to achieve target concentrations (50 or 1250 ng L^–1^) and then incubated in the dark for 7–10 days.

### Estrogen Extraction

Before each time point, microcosm bottles were capped, inverted once to mix, and allowed to settle for 30–60 min. Time-point samples were taken from 2 to 3 cm below the microcosm water surface using dedicated solvent-cleaned volumetric pipets. At each time point, 50 mL of water was transferred to a clean 250 mL amber glass bottle and spiked with 10 μL of internal standard (E2-*d4*; 0.526 ng μL^–1^ in MeOH) and then inverted five times to mix. Estrogens were extracted with solid-phase extraction cartridges (Phenomenex; Strata-X; 33 μm; 200 mg) previously conditioned with MeOH and ultrapure water. After dropwise elution using ethyl acetate, extracts were blown to dryness under N_2_ (40 °C) and reconstituted into 100 μL of 70:30 ultrapure water/MeOH. Extracts were stored at −20 °C until analysis via LC–MS/MS based on a previously validated method.^13^ Since samples were not filtered prior to SPE, reported concentrations include estrogens present in dissolved and colloidal phases.^39−41^ Solid phase concentrations were not analyzed due to the high uncertainty associated with quantifying estrogens sorbed to microcosm sediments at ng kg^–1^ levels.

### Ancillary Measurements

Additional water was removed from each microcosm at selected time points to measure temperature, pH, dissolved oxygen, total ATP (unfiltered), extracellular ATP (0.1 μm filtered), dissolved organic carbon (DOC), specific UV absorbance (SUVA_254_), nutrients (ammonium, nitrate, nitrite, phosphate, and sulfate), anions (chloride, bromide, and fluoride), and total suspended solids.

Microbial community dynamics were characterized using 16S rRNA gene sequences extracted from sediments and water filtered at 3 and 0.22 μm. Willamette River communities were characterized using water filtered on site, transported on dry ice, and stored at −80 °C. Microcosm communities were characterized over time using 400 mL samples from replicate microcosms that contained river water and sediment and had been spiked with E2. Detailed descriptions of all ancillary methods are provided in the Supporting Information.

### LC–MS/MS Analytical Method

Estrogen extracts were analyzed at the Oregon State University Mass Spectrometry Center with a high-performance liquid chromatograph (Shimadzu LC20AD) coupled to a linear ion trap triple quadrupole mass spectrometer (Applied Biosystems MDS Sciex 4000) operated using negative mode electrospray ionization. Injection volumes of 10 μL were used for all samples. Separation was achieved at 40 °C on a phenyl-hexyl or EC-C18 column (Agilent Poroshell 120; 2.1 × 50 mm; 2.7 μm) with a 0.2 μm titanium pre-column filter (Restek). The mobile phase consisted of 1 mM ammonium fluoride in deionized water (A) and methanol (B), and the gradient was linear over 10 min, from 40 to 90% B, at a flow rate of 0.350 mL min^–1^. Selected reaction monitoring (SRM) was used for quantitation, and all peak areas were normalized using the deuterated internal standard (E2-*d4*). Quantitation and confirmation ions (Table S4) were analyzed independently, and the data were validated using authentic standards according to established criteria for acceptable variability in retention time and quantitation/confirmation ion ratios.^42^

### Data Modeling Approach

At each time point, internal standard normalized peak area ratios (*R*_t_) were normalized to time zero (*R*_t_/*R*_0_) and fit according to the models described below to yield corresponding biodegradation rate constants (*k*_bio_) and half-lives (*t*_1/2,bio_). Estrogen removals from river water microcosms were modeled according to simple first order kinetics
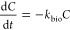
1where *R*_t_/*R*_0_ served as a proxy for estrogen concentration (*C*).

**Figure 1:**
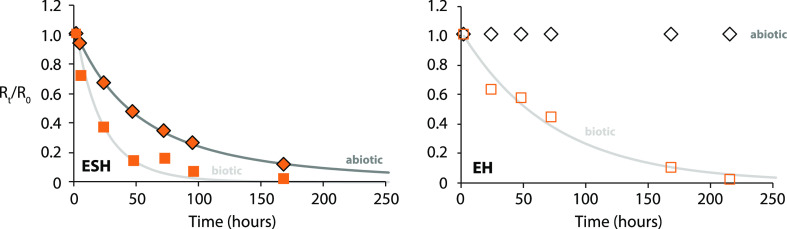
Representative estrogen kinetics for biotic (squares) and abiotic (diamonds) river water microcosms (BD-1907) with sediment (ESH; filled symbols) and without sediment (EH; open symbols) spiked with E2 at 1250 ng L^–1^ after normalization to the internal standard, time zero, and the abiotic control (EH only). Modeled fits (shown in gray) were determined by non-linear least squares regression according to the procedure described in the Supporting Information. Standard errors for modeled parameters are presented in Tables 1 and S5.

A different approach was required for microcosms containing sediment since biodegradation and abiotic processes (e.g., sorption) both contributed to the observed removals. Estrogen data from biotic microcosms containing sediment were modeled using a general *n*th-order term for the combined abiotic processes, and biodegradation was treated as a first-order process
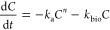
2where *k*_a_ is a lumped abiotic rate constant. Data from abiotic control microcosms containing sediment were fit according to
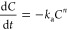
3and the modeled parameters, *k*_a_ and *n*, were then used to fit the biotic sediment microcosm data (eq 2) and extract a corresponding *k*_bio_. Best-fit curves, parameter estimates, and errors were computed by non-linear least squares regression analysis in MATLAB using the *fitnlm* function. A representative data set, including modeled fits, is shown in Figures 1 and S2. Plots and fits for all conditions during BD-1906 and BD-1907 are shown in Figures S3 and S4. Derivations of the analytical solutions to eqs 1–3 are described in detail in the Supporting Information.

## Results and Discussion

### Microcosm Characteristics and Trends

In general, chemical conditions within microcosms were similar to those of the Willamette River at the time of sample collection. During each experiment, average microcosm temperatures (19.7–21.3 °C), dissolved oxygen levels (6.1–9.4 mg L^–1^), and pH (6.8–8.0) (Figures S5 and S6) remained close to in situ river values. Likewise, concentrations of chloride, fluoride, bromide, sulfate, phosphate, and DOC were similar to the source river water (see Supporting Information for details). Calculated SUVA_254_ (3.15 L mg C^–1^ m^–1^) points to DOC with ∼24% aromatic content.^43^ In the BD-1906 and BD-1907 microcosms, NH_3_–N concentrations increased over time while NO_3_/NO_2_–N decreased (Figure S7). Both of these nitrogen pools remained well below 1 mg L^–1^ in the sediment microcosms and below 0.2 mg L^–1^ for the river water microcosms.

Representative concentrations of total suspended solids were 2.5 mg L^–1^ in river water microcosms and 25.7 mg L^–1^ in those containing sediment. Microcosm solid-water ratios and sediment porosities averaged 0.016 ± 0.04 kg L^–1^ and 0.58 ± 0.16, respectively (Table S2). Equilibrium partitioning to sediments was estimated using measured solid-water ratios, observed pH values, conservative estimates of sediment organic carbon content (1% OC), modeled values of p*K*_a_, and literature values of *K*_oc_ (Table S1). These calculations (see Supporting Information for details) suggest that at most 20% (E1; E2; monoBrE2) to 50% (diBrE2; diClE2) of the total estrogen spiked into each microcosm would be expected to sorb to sediments at equilibrium.

### Microbial Activity and Community Dynamics

The behavior of proxies for microbial activity (DO, pH, and ATP) is consistent with primarily aerobic microbial communities that increase in activity over the first 48 h, following the estrogen spike, and then slowly decline (Figures S5 and S6). The presence of sediment in river water microcosms enhanced microbial activity as measured by ATP. In biotic microcosms, ATP concentrations in 0.1 μm filtered water ranged from 0.03–0.5 nM, indicating that most of the ATP measured in biotic microcosms was contained within intact cells. Analysis of 16S rRNA data suggests that the microbial diversity of river samples and microcosms was initially similar but changed over the course of the experiment as *Alpha-* and *Gamma-proteobacteria* became more dominant (Figure S8). The abundance of known estrogen-degrading organisms (e.g., *Phyllobacterium* and *Pseudomonas*) and methylotrophs (e.g., *Methylobacterium*, *Methylophilus*, and *Methylotenera*) in our microcosms as well as their trends overtime (Figure S9) suggest that a portion of the observed microbial activity may be related to the metabolism of estrogens and the co-solvent methanol. Phyla involved in nitrification (e.g., *Nitrospinota*, *Nitrospirota*, and *Planctomycetota*) were present at low relative abundance, suggesting no more than a minor role for ammonium or nitrite oxidation in our system.

### Controls

Small-scale control experiments showed that additions of estrogen and/or co-solvent (MeOH) had no discernible impact on ATP dynamics in river water-sediment microcosms (Figures S10 and S11). Additionally, calculated biodegradation half-lives for E2 were similar whether spiked by itself or as a mixture with halogenated estrogens (Figure S3). Data from control microcosms that were not spiked with estrogen confirmed that (a) pre-spike estrogen concentrations in microcosms were below detection limits,^13^ (b) desorption from sediment over the course of the experiments was undetectable, and (c) cross-contamination between microcosms during sampling and processing was not occurring.

While microbial activity was strongly inhibited in abiotic controls containing sodium azide, we observed small residual ATP concentrations between 0.1 and 0.5 nM (Figures S5 and S6) in these microcosms. It is possible that some low-level ATP production is driven by microbes capable of respiring in the presence of azide, such as fungi and certain Gram-positive bacteria.^44−46^ If so, the relatively steady low ATP levels may be related to the fact that azide is known to inhibit only some ATP synthases.^47^ Although we did not sequence samples from azide-poisoned controls, it is possible that Gram-positive bacteria (e.g., *Actinobacteriota*) present at time zero (Figure S8) may have persisted in our azide controls over the 250 h time course.^46^

Changes in microcosm geochemistry and cell lysis due to azide additions are known to be small compared to thermal and irradiation-based sterilization methods.^45,48^ During method development, we found that microcosms inactivated by autoclaving had DOC levels that were over 10-fold higher than untreated and azide-treated microcosms. We also observed that E2 levels were stable over the course of a 250 h exposure to oxygen-saturated deionized water containing sodium azide (38 mM), which strongly suggests that direct degradation of estrogens by azide was not occurring.

### Estrogen Biodegradation Kinetics

**Figure 2:**
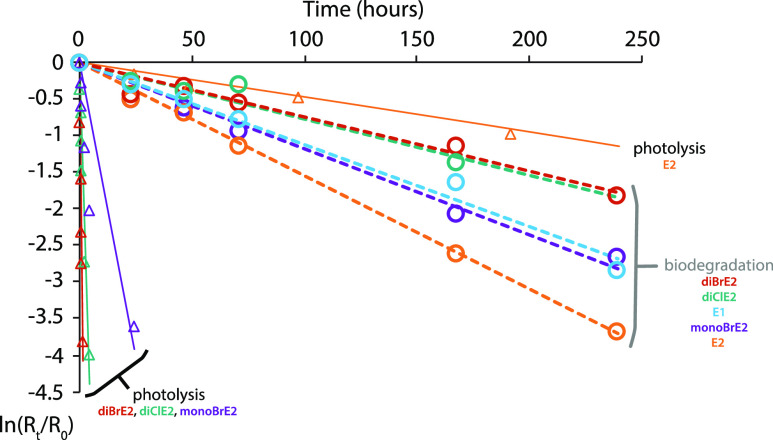
Estrogen biodegradation (BD-1906) in river water-only microcosms spiked at 50 ng L^–1^ after normalization to the internal standard, time zero, and the abiotic control. Photolysis kinetics were determined under ambient solar irradiance on 21 May–14 June 2018 using filtered (0.45 μm) water from the Willamette River (pH 7.6) spiked with individual estrogens (1 mg L^–1^; no co-solvent). These photolysis data are presented in the Supporting Information along with a description of the effects of initial concentration, tube geometry, and light screening in river water. Photolysis methods are described in detail in Milstead et al. (2018).^16^

Biodegradation rate constants and their corresponding half-life values were determined using a modeling approach that corrected for abiotic processes, such as sorption and oxidation (see Supporting Information). The average biodegradation half-life of E2 was 49 ± 16 h in river water alone and 22 ± 4 h in river water with sediment (Table 1). The corresponding half-lives for E1 (74 ± 16 and 25 ± 9 h) suggest that E1 biodegrades more slowly than E2 (Figure 2; Table 1), a finding that is supported by several studies^8,20,49−52^ but inconsistent with some others.^7,19,25,27,28^ It is clear that the presence of sediment increases biodegradation rates significantly for E1 and E2 (Table 1; Figures S3 and S4). Bradley et al.^53^ also observed faster degradation of E2 in the presence of sediment, but their use of mineralization as an end point confounds efforts to make direct quantitative comparisons to the half-lives measured here.

**Table 1:** Free and Halogenated Estrogen Biodegradation Rate Constants (*k*_bio_) and Half-Lives in River Microcosm Experiments^a^

	estrogen	microcosm bottle	initial conditions	biodegradation rate constant (*k*_bio_) (h^–1^)	biodegradation half-life (*t*_1/2,bio_) (h)
**BD-1805**					
	E2	A,B,C	50 ng L^–1^; RW; sediment	0.026 ± 0.011	26 ± 11
	E1^b^	A,B,C	50 ng L^–1^; RW; sediment	0.020 ± 0.006^b^	35 ± 11^b^
**BD-1810**					
	E2	A,B,C	50 ng L^–1^; RW; sediment	0.040 ± 0.016	17 ± 7
	E1^b^	A,B,C	50 ng L^–1^; RW; sediment	0.045 ± 0.004^b^	15 ± 1^b^
**BD-1906**					
	E2	E	50 ng L^–1^; RW	0.0167 ± 0.0012	41.5 ± 3.0
	monoBrE2	E	50 ng L^–1^; RW	0.0142 ± 0.0014	49 ± 5
	diBrE2	E	50 ng L^–1^; RW	0.0078 ± 0.0011	88 ± 12
	diClE2	E	50 ng L^–1^; RW	0.0070 ± 0.0011	98 ± 16
	E1^b^	E	50 ng L^–1^; RW	0.0075 ± 0.0006^b^	93 ± 8^b^
	diBrE1^b^	E	50 ng L^–1^; RW	0.0062 ± 0.0005^b^	112 ± 10^b^
	E2	A	50 ng L^–1^; RW	0.0170 ± 0.0006	40.9 ± 1.6
	E1^b^	A	50 ng L^–1^; RW	0.0105 ± 0.0008^b^	66 ± 5^b^
	E2	B	50 ng L^–1^; RW	0.0188 ± 0.0004	36.9 ± 0.8
	E1^b^	B	50 ng L^–1^; RW	0.0047 ± 0.0005^b^	148 ± 16^b^
	E1	F	50 ng L^–1^; RW	0.0110 ± 0.0005	63.0 ± 2.6
	E1	S	50 ng L^–1^; RW; sediment	0.0416 ± 0.0026	16.7 ± 1.1
**BD-1907**					
	E2	ESH	1250 ng L^–1^; RW; sediment	0.029 ± 0.006	24 ± 5
	monoBrE2	ESH	1250 ng L^–1^; RW; sediment	nd	nd
	diBrE2	ESH	1250 ng L^–1^; RW; sediment	nd	nd
	diClE2	ESH	1250 ng L^–1^; RW; sediment	nd	nd
	E1^b^	ESH	1250 ng L^–1^; RW; sediment	0.0294 ± 0.0028^b^	23.6 ± 2.3^b^
	diBrE1^b^	ESH	1250 ng L^–1^; RW; sediment	0.017 ± 0.003^b^	42 ± 8^b^
	E2	ESL	50 ng L^–1^; RW; sediment	0.037 ± 0.016	19 ± 8
	monoBrE2	ESL	50 ng L^–1^; RW; sediment	nd	nd
	diBrE2	ESL	50 ng L^–1^; RW; sediment	nd	nd
	diClE2	ESL	50 ng L^–1^; RW; sediment	nd	nd
	E1^b^	ESL	50 ng L^–1^; RW; sediment	0.021 ± 0.004^b^	33 ± 6^b^
	diBrE1^b^	ESL	50 ng L^–1^; RW; sediment	0.009 ± 0.004^b^	75 ± 33^b^
	E2	EH	1250 ng L^–1^; RW	0.0136 ± 0.0013	51 ± 5
	monoBrE2	EH	1250 ng L^–1^; RW	0.0145 ± 0.0019	48 ± 6
	diBrE2	EH	1250 ng L^–1^; RW	0.014 ± 0.003	50 ± 12
	diClE2	EH	1250 ng L^–1^; RW	0.0123 ± 0.0029	56 ± 13
	E1^b^	EH	1250 ng L^–1^; RW	nd	nd
	diBrE1^b^	EH	1250 ng L^–1^; RW	nd	nd
	E2	EL	50 ng L^–1^; RW	0.0092 ± 0.0004	75 ± 3
	monoBrE2	EL	50 ng L^–1^; RW	0.0072 ± 0.0008	96 ± 11
	diBrE2	EL	50 ng L^–1^; RW	0.0053 ± 0.0007	131 ± 17
	diClE2	EL	50 ng L^–1^; RW	0.0047 ± 0.0007	148 ± 21
	E1^b^	EL	50 ng L^–1^; RW	nd	nd
	diBrE1^b^	EL	50 ng L^–1^; RW	nd	nd

a Abiotic microcosm model fit parameters, including reaction order (*n*), lumped abiotic rate constants (*k*_a_), and standard errors, are included in Table S5. Uncertainties were calculated by a non-linear model fit (*fitnlm*) function in MATLAB and represent ±1 standard error.

b Biodegradation rate constants of transformation products were determined according to a kinetic model that assumed concurrent formation and decay (see Supporting Information). nd = Not determined due to low abundance peak areas, an inability to extract biodegradation rate constants when abiotic and biotic kinetics were similar, or transformation product data that were not well described by the simple formation/decay model.

Across a range of river water sources and experimental conditions, Jurgens et al.^25^ observed E2 half-lives spanning 4.8–209 h, which was attributed, in part, to the effects of nutrients and temperature on microbial activity. The only experiment conducted under river water conditions (50 ng L^–1^; 20 °C) similar to ours found an E2 biodegradation half-life of 36 h,^25^ which is within the range of those reported here. Despite large differences in flow rate, season, sediment, and microbial activity, the relative standard deviation of E2 biodegradation half-lives in Willamette River microcosms was only 32% for river water, 20% when sediment was present, and 50% overall. Ultimately, our ability to use microcosms as proxies for natural systems depends on a strong understanding of the extent to which estrogen biodegradation rates are sensitive to a range of factors, including temperature, initial concentrations, sediment characteristics, and microbial community structure.

In river water microcosms spiked with estrogens at 50 ng L^–1^, we found that estrogen biodegradation rates slowed significantly as the extent of halogenation increased (Figure 2). Under these conditions, monoBrE2, diBrE2, and diClE2 exhibited average biodegradation half-lives that were 1.5, 2.3, and 2.6-fold longer than E2, respectively (Table 1). This trend could reflect the fact that halogen substituents interfere with one or more estrogen biodegradation pathways involving the aromatic ring.^25,53^ Alternatively, greater sorption of halogenated estrogens onto river colloids^15,40,54−57^ may provide a protective effect that slows biodegradation. Based on calculated half-lives and associated uncertainties, the trend was less clear at 1250 ng L^–1^ (Table 1), which may reflect a change in the availability of sorption sites on colloids or the relative balance of degradation pathways at different initial estrogen concentrations.

**Figure 3:**
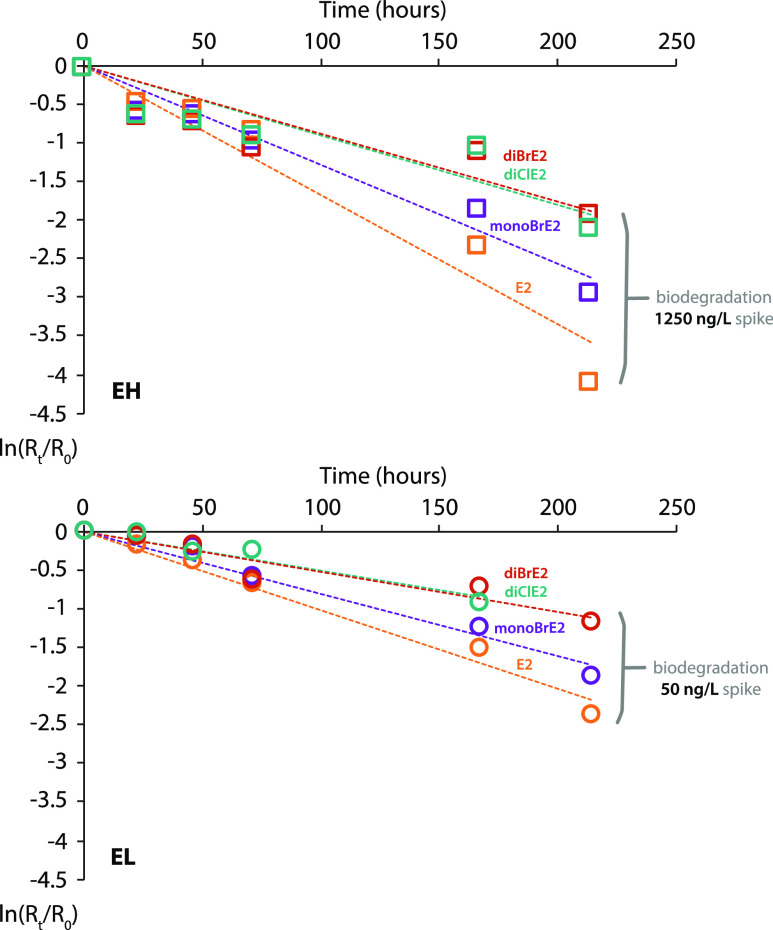
Free and halogenated estrogen biodegradation (BD-1907) in river water-only microcosms spiked at 1250 ng L^–1^ (EH) and 50 ng L^–1^ (EL) after normalization to the internal standard, time zero, and the abiotic control.

In microcosms containing sediment, E2 biodegradation half-lives were statistically indistinguishable at low (19 ± 8 h) and high (24 ± 5 h) estrogen spike conditions. Xu et al.^35^ and Li et al.^31^ observed similar behavior for free estrogens across a wide range of initial concentrations (30 ng L^–1^ to 50 μg L^–1^) in microcosms containing activated sludge. At even higher concentrations (100 μg L^–1^ to 300 mg L^–1^), others have seen slower biodegradation at higher spiking levels,^35,36,58^ a trend that was attributed to inhibitory processes and/or the scarcity of certain co-metabolic substrates. In contrast, for those microcosms containing only river water, biodegradation was slower at lower initial estrogen concentrations (Figure 3). For example, E2 degradation was 1.5× slower at 50 ng L^–1^ (*t*_1/2,bio_ = 75 ± 3 h) compared to 1250 ng L^–1^ (*t*_1/2,bio_ = 51 ± 5 h). The halogenated estrogens showed similar behavior, degrading 2.0-fold (monoBrE2) and 2.6-fold (diBrE2; diClE2) slower at 50 ng L^–1^. Slower biodegradation of free estrogens at lower initial concentrations was observed by Ke et al.,^59^ who used single strains of estrogen-degrading bacteria at estrogen levels between 50 and 2000 μg L^–1^. It is possible that the concentration effect we observed in river water-only microcosms may reflect greater contributions from bacteria that use estrogens as growth substrates, such as *Phyllobacterium* and *Sphingomonas*,^38,60−62^ while co-metabolic degradation by microbes growing on organic carbon^63−66^ may have partially masked this effect in microcosms containing sediment.

### Transformation Products and Mass Balance

**Figure 4:**
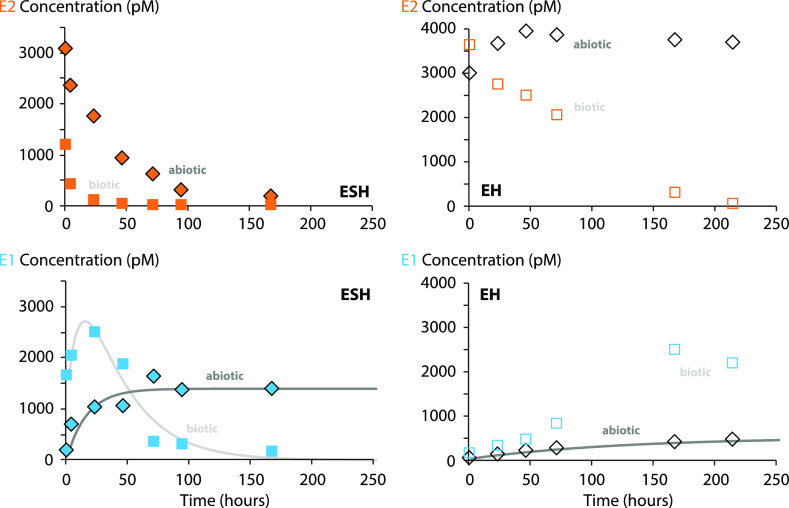
Representative estrogen kinetics (BD-1907) for biotic (squares) and abiotic (diamonds) river water microcosms with sediment (ESH; filled symbols) and without sediment (EH; open symbols) spiked with E2 at 1250 ng L^–1^. The concurrent growth and decay of E1 (blue), a transformation product of E2 (orange), was modeled according to the procedure described in the Supporting Information. Similar behavior was observed for diBrE2, including the growth and decay of diBrE1 (data not shown). Quantitation employed an internal standard (E2-*d4*) normalized calibration approach.

A wide range of E2 biotransformation products have been reported in the literature, including E1,^25,67,68^ estrogen conjugates,^69^ hydroxylated derivatives,^61^ dehydrated forms,^70^ oligomers,^71^ and ring-cleavage products.^25,53,72^ We observed that E2 and diBrE2 were biodegraded into the corresponding E1 forms (i.e., E1 and diBrE1), which were further degraded, though at a slower rate (Figure 4). The growth and subsequent decay of E1 have been observed in a range of natural and engineered environments^8,25,29,36,37,59,64,73^ and was a first-generation biodegradation intermediate predicted by the University of Minnesota Pathway Prediction System (UM-PPS).^74,75^ Other predicted E2 biotransformation intermediates included the 2-hydroxy, 4-hydroxy, and A-ring cleavage products. Since the oxidation of E2 to E1 occurs far from the aromatic ring where bromine and chlorine atoms are attached in halogenated estrogens, and given that we observed that diBrE1 followed similar growth-decay behavior during diBrE2 biodegradation, it is likely that monoBrE1 and diClE1 were also formed as intermediates during the biodegradation of the corresponding halogenated E2 forms. Although these halogenated E1 transformation products were predicted by UM-PPS, our analytical method was not suited to detecting all of them since authentic standards and expected fragmentation information were not available.

Mass balance calculations reveal that 40–80% of the parent estrogen (e.g., E2 and diBrE2) was transformed to the E1 derivative (e.g., E1 and diBrE1) in river water microcosms with and without sediment present. Others have observed similar conversion efficiencies for the biodegradation of E2 to E1 by bacteria in sewage^29,76^ and natural sediment/soil systems.^26,50^ The portion not converted to E1 may be sorbed to solids, abiotically transformed, or biodegraded via ring-cleavage pathways. Conjugated estrogen intermediates were not detected using a sensitive LC–MS/MS method^13^ and are thus unlikely transformation products. Since microcosm conditions were not suitable for either nitrification^70^ or laccase-mediated oligomerization,^71^ dehydrated estrogens and oligomeric biodegradation products were not expected.

Biodegradation half-lives of select transformation products (e.g., E1 and diBrE1) were approximated by non-linear regression of simple parent-daughter growth/decay kinetics (see Supporting Information). These data (Table 1) provide additional support for the idea that E1 forms degrade more slowly than E2 forms and that halogenated estrogens degrade more slowly than the corresponding free estrogens. Others have taken a similar approach to modeling E1 dynamics as a combination of formation from E2 degradation and removal by E1 degradation.^49,52,69,77^ While many studies have also investigated the interactions between estrogens and sediments,^12,15,78−82^ the mechanisms by which aqueous phase E1 behavior is influenced by sorption/desorption dynamics remain largely uncharacterized.

### Abiotic Processes

Estrogen removals in abiotic microcosms were enhanced by the presence of sediment (Figures 4 and S4) due to a combination of sorption and abiotic oxidation. Others have shown that free estrogen sorption to soils, sediments, and colloids is correlated to OC content^12,26,73^ and may target a small number of preferred sites first,^81,83^ reaching equilibrium on timescales of minutes to days depending on the system and the nature of the sorbent.^12,26,55,81,84^ Equilibrium partitioning calculations indicate that a maximum of 20–50% of the initial estrogen spike would sorb to sediments in our microcosms (see Supporting Information). Yet, estrogen removals in abiotic microcosms containing sediment approached 80–90%, far higher than if sorption alone were responsible.

The growth of E1 and diBrE1 in azide-poisoned microcosms (Figure 4) suggests that abiotic oxidation reactions play a role. Previous studies have documented abiotic estrogen oxidation by autoclaved sediments and soils^50,69^ and manganese oxides,^9,85,86^ as well as oligomerization by Fe^3+^-saturated montmorillonite^10^ at relevant timescales. Powder XRD and petrographic analyses suggest that our microcosm sediments contained ∼10% clay minerals, including phyllosilicates (smectite, chlorite, or vermiculite), as well as trace amounts (<1%) of hematite. These mineral phases and humic acids are known to contain redox-active iron^87−89^ and could be responsible for some portion of the observed abiotic oxidation of E2 and diBrE2.

Several other processes were less likely. Extensive abiotic oxidation by reactive oxygen species (ROS) such as hydroxyl radicals was unlikely given the quenching ability of azide and methanol^90−92^ and the fact that E1 and diBrE1 were stable in abiotic microcosms (Figure 4). The stability of E1 also rules out significant oxidation by azide-resistant fungi and Gram-positive bacteria, which degrade both E1 and E2.^7,61^ Finally, abiotic nitration reactions were negligible since NO_3_/NO_2_–N concentrations were at least 2 orders of magnitude lower than would be necessary.^93^ Therefore, we hypothesize that sorption to sediments and abiotic oxidation reactions on minerals and natural organic matter (NOM) were the main drivers of estrogen removal in our abiotic microcosms.

### Environmental Implications

In river water microcosms, biodegradation and abiotic oxidation of E2 and its halogenated forms took place on timescales of days and primarily yielded E1 derivatives, which are slightly less estrogenic than E2. Mass balance calculations indicate that biodegradation was responsible for 86% of the observed oxidation of E2 to E1 in biotic microcosms containing only river water. However, in the presence of sediment, abiotic oxidation reactions became the dominant process (71%) responsible for E1 formation. We found a similar trend for diBrE2 oxidation to diBrE1, which suggests that monoBrE2 and diClE2 could also be oxidized to their corresponding E1 derivatives.

In contrast, the photolysis of halogenated estrogens in river water occurred on sub-hour timescales. Together, these findings suggest that, in sediment-laden river water, the dominant estrogen removal processes are likely to be sorption, biodegradation, and oxidation reactions on mineral surfaces and NOM. However, in sunlit waters and near-surface environments, biodegradation of free estrogens or photolysis of halogenated forms should dominate.

Ultimately, the fate of estrogens in sewage-impacted rivers will be strongly dependent on the relative abundance of halogenated forms in wastewater effluent and the extent to which these forms are exposed to natural sunlight or UV light during wastewater treatment and in receiving waters.

## Supporting Information Available

Selected properties of free and halogenated estrogens; river water and sediment characteristics for microcosm biodegradation experiments; microcosm labels and conditions during river water biodegradation experiments; estrogen LC-ESI(−)-MS/MS transitions; abiotic model fit parameters; estrogen structures, chemical names, and abbreviations used; illustrated summary of modeling approach; estrogen degradation plots; microcosm ATP, DO, and pH trends; nitrogen-based nutrient trends; microbial communities identified using 16S rRNA gene sequencing; small-scale microcosm experiments; representative ATP calibration curve; ancillary methods and results; sorption estimates; estrogen photolysis kinetics in river water (rate constants, quantum yields, concentration dependence, and corrections under field conditions); and modeling approach and derivations (PDF)

## References

[ref1] Caldwell D. J.; Mastrocco F.; Anderson P. D.; Länge R.; Sumpter J. P. Predicted-no-effect concentrations for the steroid estrogens estrone, 17β-estradiol, estriol, and 17α-ethinylestradiol. Environ. Toxicol. Chem. 2012, 31, 1396–1406. 10.1002/etc.1825. 22488680

[ref2] Pereira A.; Silva L.; Laranjeiro C.; Lino C.; Pena A. Selected pharmaceuticals in different aquatic compartments: Part II-Toxicity and environmental risk assessment. Molecules 2020, 25, 1796 10.3390/molecules25081796. 32295269 PMC7221825

[ref3] Matthiessen P.; Wheeler J. R.; Weltje L. A review of the evidence for endocrine disrupting effects of current-use chemicals on wildlife populations. Crit. Rev. Toxicol. 2018, 48, 195–216. 10.1080/10408444.2017.1397099. 29171327

[ref4] Khanal S. K.; Xie B.; Thompson M. L.; Sung S. W.; Ong S. K.; Van Leeuwen J. Fate, transport, and biodegradation of natural estrogens in the environment and engineered systems. Environ. Sci. Technol. 2006, 40, 6537–6546. 10.1021/es0607739. 17144275

[ref5] Shore L. S.; Shemesh M. In Naturally produced steroid hormones and their release into the environment, Symposium on Implications of Endocrine Active Substances for Humans and Wildlife, Yokohama, Japan, Nov 17–21, 2002; International Union of Pure and Applied Chemistry: Yokohama, Japan, 2002; pp 1859-1871.

[ref6] Griffith D. R.; Kido Soule M. C.; Eglinton T. I.; Kujawinski E. B.; Gschwend P. M. Steroidal estrogen sources in a sewage-impacted coastal ocean. Environ. Sci.: Processes Impacts 2016, 18, 981–991. 10.1039/c6em00127k. 27465804

[ref7] Zhao X.; Grimes K. L.; Colosi L. M.; Lung W. S. Attenuation, transport, and management of estrogens: A review. Chemosphere 2019, 230, 462–478. 10.1016/j.chemosphere.2019.05.086. 31121510

[ref8] Ma L.; Yates S. R. Degradation and metabolite formation of 17ß-estradiol-3-glucuronide and 17ß-estradiol-3-sulphate in river water and sediment. Water Res. 2018, 139, 1–9. 10.1016/j.watres.2018.03.071. 29621712

[ref9] Daniel Sheng G.; Xu C.; Xu L.; Qiu Y.; Zhou H. Abiotic oxidation of 17β-estradiol by soil manganese oxides. Environ. Pollut. 2009, 157, 2710–2715. 10.1016/j.envpol.2009.04.030. 19467566

[ref10] Qin C.; Troya D.; Shang C.; Hildreth S.; Helm R.; Xia K. Surface catalyzed oxidative oligomerization of 17β-estradiol by Fe3+-saturated montmorillonite. Environ. Sci. Technol. 2015, 49, 956–964. 10.1021/es504815t. 25496116

[ref11] Tan D. T.; Temme H. R.; Arnold W. A.; Novak P. J. Estrone degradation: Does organic matter (quality), matter?. Environ. Sci. Technol. 2015, 49, 498–503. 10.1021/es504424v. 25454582

[ref12] Lai K. M.; Johnson K. L.; Scrimshaw M. D.; Lester J. N. Binding of waterborne steroid estrogens to solid phases in river and estuarine systems. Environ. Sci. Technol. 2000, 34, 3890–3894. 10.1021/es9912729.

[ref13] Griffith D. R.; Kido Soule M. C.; Matsufuji H.; Eglinton T. I.; Kujawinski E. B.; Gschwend P. M. Measuring free, conjugated, and halogenated estrogens in secondary treated wastewater effluent. Environ. Sci. Technol. 2014, 48, 2569–2578. 10.1021/es402809u. 24476066

[ref14] Nakamura H.; Kuruto-Niwa R.; Uchida M.; Terao Y. Formation of chlorinated estrones via hypochlorous disinfection of wastewater effluent containing estrone. Chemosphere 2007, 66, 1441–1448. 10.1016/j.chemosphere.2006.09.011. 17081588

[ref15] Casey F. X. M.; Shappell N. W.; Hakk H. Halogenated 17β-estradiol surrogates: Synthesis, estrogenic activity, and initial investigations of fate in soil/water systems. J. Environ. Qual. 2017, 46, 802–810. 10.2134/jeq2017.02.0053. 28783794

[ref16] Milstead R. P.; Nance K. T.; Tarnas K. S.; Egelhofer K. E.; Griffith D. R. Photochemical degradation of halogenated estrogens under natural solar irradiance. Environ. Sci.: Processes Impacts 2018, 20, 1350–1360. 10.1039/c8em00275d. 30211921

[ref17] Fischer K.; Majewsky M. Cometabolic degradation of organic wastewater micropollutants by activated sludge and sludge-inherent microorganisms. Appl. Microbiol. Biotechnol. 2014, 98, 6583–6597. 10.1007/s00253-014-5826-0. 24866947

[ref18] Yu C. P.; Deeb R. A.; Chu K. H. Microbial degradation of steroidal estrogens. Chemosphere 2013, 91, 1225–1235. 10.1016/j.chemosphere.2013.01.112. 23517889

[ref19] Carr D. L.; Morse A. N.; Zak J. C.; Anderson T. A. Microbially mediated degradation of common pharmaceuticals and personal care products in soil under aerobic and reduced oxygen conditions. Water, Air, Soil Pollut. 2011, 216, 633–642. 10.1007/s11270-010-0558-y.

[ref20] Xuan R.; Blassengale A. A.; Wang Q. Degradation of estrogenic hormones in a silt loam soil. J. Agric. Food Chem. 2008, 56, 9152–9158. 10.1021/jf8016942. 18778070

[ref21] Ying G. G.; Kookana R. S. Degradation of five selected endocrine-disrupting chemicals in seawater and marine sediment. Environ. Sci. Technol. 2003, 37, 1256–1260. 10.1021/es0262232.

[ref22] Ying G. G.; Kookana R. S. Sorption and degradation of estrogen-like-endocrine disrupting chemicals in soil. Environ. Toxicol. Chem. 2005, 24, 2640–2645. 10.1897/05-074r.1. 16268167

[ref23] Ying G. G.; Toze S.; Hanna J.; Yu X. Y.; Dillon P.; Kookana R. S. Decay of endocrine-disrupting chemicals in aerobic and anoxic groundwater. Water Res. 2008, 42, 1133–1141. 10.1016/j.watres.2007.08.029. 17897695

[ref24] Ying G. G.; Kookana R. S.; Dillon P. Sorption and degradation of selected five endocrine disrupting chemicals in aquifer material. Water Res. 2003, 37, 3785–3791. 10.1016/s0043-1354(03)00261-6. 12867347

[ref25] Jurgens M. D.; Holthaus K. I. E.; Johnson A. C.; Smith J. J. L.; Hetheridge M.; Williams R. J. The potential for estradiol and ethinylestradiol degradation in English rivers. Environ. Toxicol. Chem. 2002, 21, 480–488. 10.1002/etc.5620210302. 11883412

[ref26] Lee L. S.; Strock T. J.; Sarmah A. K.; Rao P. S. C. Sorption and dissipation of testosterone, estrogens, and their primary transformation products in soils and sediment. Environ. Sci. Technol. 2003, 37, 4098–4105. 10.1021/es020998t. 14524441

[ref27] Das B. S.; Lee L. S.; Rao P. S. C.; Hultgren R. P. Sorption and degradation of steroid hormones in soils during transport: column studies and model evaluation. Environ. Sci. Technol. 2004, 38, 1460–1470. 10.1021/es034898e. 15046348

[ref28] Robinson J. A.; Ma Q.; Staveley J. P.; Smolenski W. J.; Ericson J. Degradation and transformation of 17Α-estradiol in water–sediment systems under controlled aerobic and anaerobic conditions. Environ. Toxicol. Chem. 2017, 36, 621–629. 10.1002/etc.3383. 26801177

[ref29] Lee H. B.; Liu D. Degradation of 17β-estradiol and its metabolities by sewage bacteria. Water, Air, Soil Pollut. 2002, 134, 353–368.

[ref30] Lee J. H.; Zhou J. L.; Lee Y.; Oh S. Y.; Kim S. D. Changes in the sorption and rate of 17β-estradiol biodegradation by dissolved organic matter collected from different water sources. J. Environ. Monit. 2012, 14, 543–551. 10.1039/c1em10690b. 22193409

[ref31] Li F.; Yuasa A.; Obara A.; Mathews A. P. Aerobic batch degradation of 17-β estradiol (E2) by activated sludge: Effects of spiking E2 concentrations, MLVSS and temperatures. Water Res. 2005, 39, 2065–2075. 10.1016/j.watres.2005.02.009. 15949528

[ref32] Li J.; Jiang L.; Liu X.; Lv J. Adsorption and aerobic biodegradation of four selected endocrine disrupting chemicals in soil-water system. Int. Biodeterior. Biodegrad. 2013, 76, 3–7. 10.1016/j.ibiod.2012.06.004.

[ref33] Liu Y.; Sun W.; Ni J. Biodegradation of bisphenol A, 17β-estradiol, and 17α- ethynylestradiol in river water. Int. J. Environ. Pollut. 2011, 45, 225–236. 10.1504/ijep.2011.039098.

[ref34] Liu Z. H.; Lu G. N.; Yin H.; Dang Z.; Rittmann B. Removal of natural estrogens and their conjugates in municipal wastewater treatment plants: A critical review. Environ. Sci. Technol. 2015, 49, 5288–5300. 10.1021/acs.est.5b00399. 25844648

[ref35] Xu N. A. N.; Johnson A. C.; Jürgens M. D.; Llewellyn N. R.; Hankins N. P.; Darton R. C. Estrogen concentration affects its biodegradation rate in activated sludge. Environ. Toxicol. Chem. 2009, 28, 2263–2270. 10.1897/08-577.1. 19572766

[ref36] Ternes T. A.; Kreckel P.; Mueller J. Behaviour and occurrence of estrogens in municipal sewage treatment plants - II. Aerobic batch experiments with activated sludge. Sci. Total Environ. 1999, 225, 91–99. 10.1016/s0048-9697(98)00335-0. 10028706

[ref37] Mashtare M. L.; Green D. A.; Lee L. S. Biotransformation of 17α- and 17β-estradiol in aerobic soils. Chemosphere 2013, 90, 647–652. 10.1016/j.chemosphere.2012.09.032. 23084590

[ref38] Zhang C.; Li Y.; Wang C.; Niu L.; Cai W. Occurrence of endocrine disrupting compounds in aqueous environment and their bacterial degradation: A review. Crit. Rev. Environ. Sci. Technol. 2016, 46, 1–59. 10.1080/10643389.2015.1061881.

[ref39] Lee J.; Cho J.; Kim S. H.; Kim S. D. Influence of 17Β-estradiol binding by dissolved organic matter isolated from wastewater effluent on estrogenic activity. Ecotoxicol. Environ. Saf. 2011, 74, 1280–1287. 10.1016/j.ecoenv.2011.02.010. 21397328

[ref40] Holbrook R. D.; Love N. G.; Novak J. T. Sorption of 17-beta-estradiol and 17 alpha-ethinylestradiol by colloidal organic carbon derived from biological wastewater treatment systems. Environ. Sci. Technol. 2004, 38, 3322–3329. 10.1021/es035122g. 15260331

[ref41] Yamamoto H.; Liljestrand H. M. The fate of estrogenic compounds in the aquatic environment: sorption onto organic colloids. Water Sci. Technol. 2003, 47, 77–84. 10.2166/wst.2003.0497. 12830944

[ref42] Li L. Y. T.; Campbell D. A.; Bennett P. K.; Henion J. Acceptance criteria for ultratrace HPLC-tandem mass spectrometry: Quantitative and qualitative determination of sulfonylurea herbicides in soil. Anal. Chem. 1996, 68, 3397–3404. 10.1021/ac960375w. 21619275

[ref43] Weishaar J. L.; Aiken G. R.; Bergamaschi B. A.; Fram M. S.; Fujii R.; Mopper K. Evaluation of specific ultraviolet absorbance as an indicator of the chemical composition and reactivity of dissolved organic carbon. Environ. Sci. Technol. 2003, 37, 4702–4708. 10.1021/es030360x. 14594381

[ref44] Cabrol L.; Quéméneur M.; Misson B. Inhibitory effects of sodium azide on microbial growth in experimental resuspension of marine sediment. J. Microbiol. Methods 2017, 133, 62–65. 10.1016/j.mimet.2016.12.021. 28039035

[ref45] Otte J. M.; Blackwell N.; Soos V.; Rughöft S.; Maisch M.; Kappler A.; Kleindienst S.; Schmidt C. Sterilization impacts on marine sediment-Are we able to inactivate microorganisms in environmental samples?. FEMS Microbiol. Ecol. 2018, 94, fiy189 10.1093/femsec/fiy189. 30247566

[ref46] K Bore E.; Apostel C.; Halicki S.; Kuzyakov Y.; Dippold M. A. Soil microorganisms can overcome respiration inhibition by coupling intra-and extracellular metabolism: 13C metabolic tracing reveals the mechanisms. ISME J. 2017, 11, 1423–1433. 10.1038/ismej.2017.3. 28157187 PMC5437355

[ref47] Bowler M. W.; Montgomery M. G.; Leslie A. G. W.; Walker J. E. How azide inhibits ATP hydrolysis by the F-ATPases. Proc. Natl. Acad. Sci. U.S.A. 2006, 103, 8646–8649. 10.1073/pnas.0602915103. 16728506 PMC1469772

[ref48] Yi T.; Harperjr W. The effect of biomass characteristics on the partitioning and sorption hysteresis of 17 α-ethinylestradiol. Water Res. 2007, 41, 1543–1553. 10.1016/j.watres.2006.12.023. 17276478

[ref49] Zheng W.; Li X.; Yates S. R.; Bradford S. A. Anaerobic transformation kinetics and mechanism of steroid estrogenic hormones in dairy lagoon water. Environ. Sci. Technol. 2012, 46, 5471–5478. 10.1021/es301551h. 22519517

[ref50] Colucci M. S.; Bork H.; Topp E. Persistence of estrogenic hormones in agricultural soils: I. 17 beta-estradiol and estrone. J. Environ. Qual. 2001, 30, 2070–2076. 10.2134/jeq2001.2070. 11790015

[ref51] Mashtare M. L.; Lee L. S.; Nies L. F.; Turco R. F. Transformation of 17α-Estradiol, 17β-estradiol, and estrone in sediments under nitrate- and sulfate-reducing conditions. Environ. Sci. Technol. 2013, 47, 7178–7185. 10.1021/es4008382. 23706021

[ref52] Joss A.; Andersen H.; Ternes T.; Richle P. R.; Siegrist H. Removal of estrogens in municipal wastewater treatment under aerobic and anaerobic conditions: Consequences for plant optimization. Environ. Sci. Technol. 2004, 38, 3047–3055. 10.1021/es0351488. 15224734

[ref53] Bradley P. M.; Barber L. B.; Chapelle F. H.; Gray J. L.; Kolpin D. W.; McMahon P. B. Biodegradation of 17 beta-estradiol, estrone and testosterone in stream sediments. Environ. Sci. Technol. 2009, 43, 1902–1910. 10.1021/es802797j. 19368190

[ref54] Bowman J. C.; Zhou J. L.; Readman J. W. Sediment-water interactions of natural oestrogens under estuarine conditions. Mar. Chem. 2002, 77, 263–276. 10.1016/s0304-4203(02)00006-3.

[ref55] Liu R.; Wilding A.; Hibberd A.; Zhou J. L. Partition of endocrine-disrupting chemicals between colloids and dissolved phase as determined by cross-flow ultrafiltration. Environ. Sci. Technol. 2005, 39, 2753–2761. 10.1021/es0484404. 15884373

[ref56] Nie M.; Yang Y.; Liu M.; Yan C.; Shi H.; Dong W.; Zhou J. L. Environmental estrogens in a drinking water reservoir area in Shanghai: Occurrence, colloidal contribution and risk assessment. Sci. Total Environ. 2014, 487, 785–791. 10.1016/j.scitotenv.2013.12.010. 24364991

[ref57] Zhou J. L.; Liu R.; Wilding A.; Hibberd A. Sorption of selected endocrine disrupting chemicals to different aquatic colloids. Environ. Sci. Technol. 2007, 41, 206–213. 10.1021/es0619298. 17265949

[ref58] Stumm-Zollinger E.; Fair G. M. Biodegradation of steroid hormones. J.—Water Pollut. Control Fed. 1965, 37, 1506–1510. 5849629

[ref59] Ke J.; Zhuang W.; Gin K. Y. H.; Reinhard M.; Hoon L. T.; Tay J. H. Characterization of estrogen-degrading bacteria isolated from an artificial sandy aquifer with ultrafiltered secondary effluent as the medium. Appl. Microbiol. Biotechnol. 2007, 75, 1163–1171. 10.1007/s00253-007-0923-y. 17396255

[ref60] Kagle J.; Porter A. W.; Murdoch R. W.; Rivera-Cancel G.; Hay A. G. Chapter 3 Biodegradation of Pharmaceutical and Personal Care Products. Advances in Applied Microbiology; Elsevier, 2009; Vol. 67, pp 65–108. 19245937 10.1016/S0065-2164(08)01003-4

[ref61] Kurisu F.; Ogura M.; Saitoh S.; Yamazoe A.; Yagi O. Degradation of natural estrogen and identification of the metabolites produced by soil isolates of Rhodococcus sp. and Sphingomonas sp. J. Biosci. Bioeng. 2010, 109, 576–582. 10.1016/j.jbiosc.2009.11.006. 20471597

[ref62] Li Z.; Nandakumar R.; Madayiputhiya N.; Li X. Proteomic analysis of 17β-estradiol degradation by Stenotrophomonas maltophilia. Environ. Sci. Technol. 2012, 46, 5947–5955. 10.1021/es300273k. 22587609

[ref63] Kim M. H.; Hao O. J. Cometabolic degradation of chlorophenols by Acinetobacter species. Water Res. 1999, 33, 562–574. 10.1016/s0043-1354(98)00228-0.

[ref64] Yu C. P.; Roh H.; Chu K. H. 17β-estradiol-degrading bacteria isolated from activated sludge. Environ. Sci. Technol. 2007, 41, 486–492. 10.1021/es060923f. 17310711

[ref65] Pauwels B.; Wille K.; Noppe H.; De Brabander H.; Van De Wiele T.; Verstraete W.; Boon N. 17α-ethinylestradiol cometabolism by bacteria degrading estrone, 17β-estradiol and estriol. Biodegradation 2008, 19, 683–693. 10.1007/s10532-007-9173-z. 18181025

[ref66] Egli T. How to live at very low substrate concentration. Water Res. 2010, 44, 4826–4837. 10.1016/j.watres.2010.07.023. 20688348

[ref67] Writer J. H.; Ryan J. N.; Keefe S. H.; Barber L. B. Fate of 4-nonylphenol and 17β-estradiol in the Redwood River of Minnesota. Environ. Sci. Technol. 2012, 46, 860–868. 10.1021/es2031664. 22208914

[ref68] Hom-Diaz A.; Llorca M.; Rodríguez-Mozaz S.; Vicent T.; Barceló D.; Blánquez P. Microalgae cultivation on wastewater digestate: β-estradiol and 17α-ethynylestradiol degradation and transformation products identification. J. Environ. Manage. 2015, 155, 106–113. 10.1016/j.jenvman.2015.03.003. 25785785

[ref69] Goeppert N.; Dror I.; Berkowitz B. Detection, fate and transport of estrogen family hormones in soil. Chemosphere 2014, 95, 336–345. 10.1016/j.chemosphere.2013.09.039. 24134891

[ref70] Nakai S.; Yamamura A.; Tanaka S.; Shi J.; Nishikawa M.; Nakashimada Y.; Hosomi M. Pathway of 17β-estradiol degradation by Nitrosomonas europaea and reduction in 17β-estradiol-derived estrogenic activity. Environ. Chem. Lett. 2011, 9, 1–6. 10.1007/s10311-010-0308-9.

[ref71] Lloret L.; Eibes G.; Moreira M. T.; Feijoo G.; Lema J. M. Removal of estrogenic compounds from filtered secondary wastewater effluent in a continuous enzymatic membrane reactor. Identification of biotransformation products. Environ. Sci. Technol. 2013, 47, 4536–4543. 10.1021/es304783k. 23544499

[ref72] Wang Y.; Sun Q.; Li Y.; Wang H.; Wu K.; Yu C. P. Biotransformation of estrone, 17β-estradiol and 17α-ethynylestradiol by four species of microalgae. Ecotoxicol. Environ. Saf. 2019, 180, 723–732. 10.1016/j.ecoenv.2019.05.061. 31152986

[ref73] Lee J. H.; Zhou J. L.; Kim S. D. Effects of biodegradation and sorption by humic acid on the estrogenicity of 17β-estradiol. Chemosphere 2011, 85, 1383–1389. 10.1016/j.chemosphere.2011.08.003. 21872903

[ref74] Gao J.; Ellis L. B. M.; Wackett L. P. The University of Minnesota Biocatalysis/Biodegradation Database: Improving public access. Nucleic Acids Res. 2009, 38, D488–D491. 10.1093/nar/gkp771. 19767608 PMC2808978

[ref75] Gao J.; Ellis L. B. M.; Wackett L. P. The University of Minnesota Pathway Prediction System: Multi-level prediction and visualization. Nucleic Acids Res. 2011, 39, W406–W411. 10.1093/nar/gkr200. 21486753 PMC3125723

[ref76] Gaulke L. S.; Strand S. E.; Kalhorn T. F.; Stensel H. D. Estrogen biodegradation kinetics and estrogenic activity reduction for two biological wastewater treatment methods. Environ. Sci. Technol. 2009, 43, 7111–7116. 10.1021/es901194c. 19806750

[ref77] Steiner L. D.; Bidwell V. J.; Di H. J.; Cameron K. C.; Northcott G. L. Transport and modeling of estrogenic hormones in a dairy farm effluent through undisturbed soil lysimeters. Environ. Sci. Technol. 2010, 44, 2341–2347. 10.1021/es9031216. 20166695

[ref78] Holthaus K. I. E.; Johnson A. C.; Jürgens M. D.; Williams R. J.; Smith J. J. L.; Carter J. E. The potential for estradiol and ethinylestradiol to sorb to suspended and bed sediments in some English rivers. Environ. Toxicol. Chem. 2002, 21, 2526–2535. 10.1002/etc.5620211202. 12463545

[ref79] Labadie P.; Cundy A. B.; Stone K.; Andrews M.; Valbonesi S.; Hill E. M. Evidence for the migration of steroidal estrogens through river bed sediments. Environ. Sci. Technol. 2007, 41, 4299–4304. 10.1021/es063062j. 17626428

[ref80] Liu D.; Lung W. S.; Colosi L. M. Effects of sorption kinetics on the fate and transport of pharmaceuticals in estuaries. Chemosphere 2013, 92, 1001–1009. 10.1016/j.chemosphere.2013.03.029. 23632244

[ref81] Yu Z.; Xiao B.; Huang W.; Peng P. Sorption of steroid estrogens to soils and sediments. Environ. Toxicol. Chem. 2004, 23, 531–539. 10.1897/03-192. 15285343

[ref82] Zhang J.; Yang G. P.; Li Q.; Cao X.; Liu G. Study on the sorption behaviour of estrone on marine sediments. Mar. Pollut. Bull. 2013, 76, 220–226. 10.1016/j.marpolbul.2013.08.038. 24054732

[ref83] Van Emmerik T.; Angove M. J.; Johnson B. B.; Wells J. D.; Fernandes M. B. Sorption of 17β -estradiol onto selected soil minerals. J. Colloid Interface Sci. 2003, 266, 33–39. 10.1016/s0021-9797(03)00597-6. 12957579

[ref84] Casey F. X. M.; Šimůnek J.; Lee J.; Larsen G. L.; Hakk H. Sorption, mobility, and transformation of estrogenic hormones in natural soil. J. Environ. Qual. 2005, 34, 1372–1379. 10.2134/jeq2004.0290. 15998860

[ref85] Xu L.; Xu C.; Zhao M. R.; Qiu Y. P.; Sheng G. D. Oxidative removal of aqueous steroid estrogens by manganese oxides. Water Res. 2008, 42, 5038–5044. 10.1016/j.watres.2008.09.016. 18929389

[ref86] Jiang L.; Huang C.; Chen J.; Chen X. Oxidative transformation of 17β-estradiol by MnO2 in aqueous solution. Arch. Environ. Contam. Toxicol. 2009, 57, 221–229. 10.1007/s00244-008-9257-8. 19002738

[ref87] Ilgen A. G.; Kukkadapu R. K.; Leung K.; Washington R. E. “Switching on” iron in clay minerals. Environ. Sci.: Nano 2019, 6, 1704–1715. 10.1039/c9en00228f.

[ref88] Page S. E.; Sander M.; Arnold W. A.; McNeill K. Hydroxyl radical formation upon oxidation of reduced humic acids by oxygen in the dark. Environ. Sci. Technol. 2012, 46, 1590–1597. 10.1021/es203836f. 22201224

[ref89] Huang J.; Jones A.; Waite T. D.; Chen Y.; Huang X.; Rosso K. M.; Kappler A.; Mansor M.; Tratnyek P. G.; Zhang H. Fe(II) Redox Chemistry in the Environment. Chem. Rev. 2021, 121, 8161–8233. 10.1021/acs.chemrev.0c01286. 34143612

[ref90] Li M. Y.; Cline C. S.; Koker E. B.; Carmichael H. H.; Chignell C. F.; Bilski P. Quenching of singlet molecular oxygen (1O2) by azide anion in solvent mixtures. Photochem. Photobiol. 2001, 74, 760–764. 10.1562/0031-8655(2001)074<0760:qosmoo>2.0.co;2. 11783930

[ref91] Guo Y.; Long J.; Huang J.; Yu G.; Wang Y. Can the commonly used quenching method really evaluate the role of reactive oxygen species in pollutant abatement during catalytic ozonation?. Water Res. 2022, 215, 118275 10.1016/j.watres.2022.118275. 35305491

[ref92] Arlos M. J.; Liang R.; Li Chun Fong L. C. M.; Zhou N. Y.; Ptacek C. J.; Andrews S. A.; Servos M. R. Influence of methanol when used as a water-miscible carrier of pharmaceuticals in TiO2 photocatalytic degradation experiments. J. Environ. Chem. Eng. 2017, 5, 4497–4504. 10.1016/j.jece.2017.08.048.

[ref93] Gaulke L. S.; Strand S. E.; Kalhorn T. F.; Stensel H. D. 17 alpha-ethinylestradiol transformation via abiotic nitration in the presence of ammonia oxidizing bacteria. Environ. Sci. Technol. 2008, 42, 7622–7627. 10.1021/es801503u. 18983084

