# Supplementary material for: Microbial Degradation of Free and Halogenated Estrogens in River Water-Sediment Microcosms
Source: Environ Sci Technol. 2023 Jul 10;57(29):10782–91. doi: 10.1021/acs.est.3c00801 (PMC10373497; doi:10.1021/acs.est.3c00801)
Supplement: Supplementary file 1 — es3c00801_si_001.pdf [file es3c00801_si_001.pdf]

Microbial degradation of free and halogenated estrogens in river water-sediment microcosms

David R. Griffith*, MacKayla Carolan, Manuel Marcos Gutierrez, Anya Romig, Nathan Garcia-Diaz, Carolyn P. Hutchinson, and Rosa León Zayas

Willamette University, 900 State Street, Salem, OR 97301, United States

**Email: dgriffit@willamette.edu*

Supporting Information

Summary:

41 pages

Cover page

SI Tables (S1, S2, S3, S4, S5)

SI Figures (S1, S2, S3, S4, S5, S6, S7, S8, S9, S10, S11, S12)

Ancillary Methods

Ancillary Results

Sorption Estimates

Photolysis

Modeling Approach and Derivations

References

Table S1. Selected properties of free and halogenated estrogens.

Estrogen Abbreviation	Molecular Weight	Aqueous Solubility (mg L^{-1})[a]	Henry's Constant (Pa m^3 mol^{-1})[a]	pK_a[b]	$logK_{ow}$[a]	$logK_{oc}$ (experimental)	$logK_{oc}$ (predicted)[a]
E1	270.37	30[c]	3.85×10^{-5}	10.77[d]	3.13[e]	3.19[f]	3.02
E2	272.39	3.9[c]	3.69×10^{-6}	10.71[d]	4.01[e]	3.24[g]	2.90
monoBrE2	351.29	5.6	1.47×10^{-6}	8.99	4.83	3.28[g]	3.35
diBrE2	430.18	0.32	5.85×10^{-7}	7.50	5.72	3.99[g]	3.84
diClE2	341.28	2.9	2.02×10^{-6}	7.43	5.23	3.99[h]	3.57

[a](US EPA 2013)[1]; [b](Hilal et al. 2003)[2]; [c](Yalkowsky and Dannenfelser 1992)[3]; [d](Lewis and Archer 1979)[4]; [e](Hansch et al. 1995)[5]; [f](Lee et al. 2003)[6]; [g](Casey et al. 2017)[7]; [h](assumes the same value as diBrE2)

Table S2. River water and sediment characteristics for microcosm biodegradation experiments.

	**BD-1805**	**BD-1810**	**BD-1906**	**BD-1907**
Sampling Date	24 May 2018	3 October 2018	30 May 2019	18 July 2019
Microcosm Experiment Dates	29 May – 5 July 2018	8 – 15 October 2018	3 – 19 June 2019	22 – 31 July 2019
Sediment Collection Site	45º 00.52' N 123º 04.24' W	45º 00.53' N 123º 04.25' W	45º 00.51' N 123º 04.25' W	45º 00.51' N 123º 04.25' W
Water Collection Site	45º 00.52' N 123º 04.30' W	45º 00.52' N 123º 04.30' W	45º 00.52' N 123º 04.30' W	45º 00.47' N 123º 04.28' W
River Depth (m)	2.4	1.8	2.0	1.2
Sampling Depth (m)	0.5	0.5	0.5	0.5
River Water Temperature (°C)	nd	16.4	14.7	20.4
River Flow[a] ($m^3\ s^{-1}$)	416	215	425	199
Sediment Porosity	0.595	0.354	0.637	0.742
Microcosm Wet Sediment Mass (g)	50	50	50	50
Microcosm Water Volume (L)	2	2	2	2
Wet Sediment Solid to Water Ratio (kg L^{-1})	1.70	4.56	1.42	0.870
Microcosm Solid to Water Ratio (kg L^{-1})	0.0157	0.0205	0.0147	0.0116

[a] Willamette river flow rate measured by USGS at the Salem, OR gauging station (14191000)
nd = Not determined.

Table S3. Summary of microcosm labels and conditions during river water (RW) biodegradation experiments, including ultrapure water (DI) controls, abiotic controls, and bottles dedicated to measuring microbial diversity and non-target transformation products.

	Estrogen	Microcosm Label	Initial Conditions	Microbial Inhibition[a]	Methanol Co-solvent[b]
**BD-1805**					
	E2	A	50 ng L^{-1}; RW; sediment		yes
	E2	B	50 ng L^{-1}; RW; sediment		yes
	E2	C	50 ng L^{-1}; RW; sediment		yes
	E2	J	50 ng L^{-1}; RW; sediment	yes	yes
	none	K	RW; sediment		
	E2	L	50 ng L^{-1}; DI		yes
	E2	X[c]	50 ng L^{-1}; RW; sediment		yes
	E2	Y[d]	50 ng L^{-1}; RW; sediment		yes
	none	Z	DI		
**BD-1810**					
	E2	A	50 ng L^{-1}; RW; sediment		yes
	E2	B	50 ng L^{-1}; RW; sediment		yes
	E2	C	50 ng L^{-1}; RW; sediment		yes
	E2	J	50 ng L^{-1}; RW; sediment	yes	yes
	none	K	RW; sediment		
	E2	X[c]	50 ng L^{-1}; RW; sediment		yes
	E2	Y[d]	50 ng L^{-1}; RW; sediment		yes
	none	Z	DI		
**BD-1906**					
	E2	A	50 ng L^{-1}; RW		yes
	E2	B	50 ng L^{-1}; RW		yes
	E1	D	50 ng L^{-1}; RW	yes	yes
	E2, monoBrE2, diBrE2, diClE2	E	50 ng L^{-1}; RW		yes
	E1	F	50 ng L^{-1}; RW		yes
	E2	J	50 ng L^{-1}; RW	yes	yes

E1	S	50 ng L^{-1}; RW; sediment		yes
E1	W	50 ng L^{-1}; RW; sediment	yes	yes
E2	X[c]	50 ng L^{-1}; RW		yes
E2	Y[d]	50 ng L^{-1}; RW		yes
none	Z	DI		
**BD-1907**				
E2, monoBrE2, diBrE2, diClE2	EL	50 ng L^{-1}; RW		yes
E2, monoBrE2, diBrE2, diClE2	EH	1250 ng L^{-1}; RW		yes
E2, monoBrE2, diBrE2, diClE2	ESL	50 ng L^{-1}; RW; sediment		yes
E2, monoBrE2, diBrE2, diClE2	ESH	1250 ng L^{-1}; RW; sediment		yes
E2, monoBrE2, diBrE2, diClE2	zEL	50 ng L^{-1}; RW	yes	yes
E2, monoBrE2, diBrE2, diClE2	zEH	1250 ng L^{-1}; RW	yes	yes
E2, monoBrE2, diBrE2, diClE2	zESL	50 ng L^{-1}; RW; sediment	yes	yes
E2, monoBrE2, diBrE2, diClE2	zESH	1250 ng L^{-1}; RW; sediment	yes	yes
E2, monoBrE2, diBrE2, diClE2	X[c]	1250 ng L^{-1}; RW		yes
E2, monoBrE2, diBrE2, diClE2	Y[d]	50 ng L^{-1}; RW		yes
none	Z	DI		

[a] Microbial activity was inhibited by sodium azide (NaN_3; 38 mM) added to abiotic control microcosms.

[b] Methanol (10 µL) was used as the delivery solvent for all estrogen spikes.

[c] Bottle X was used for to microbial diversity measurements via 16S rRNA gene sequencing.

[d] Bottle Y was used for non-target transformation product analysis by LC-QTOF-MS. Data are not shown since instrumental sensitivity was not sufficient to identify unknown transformation products when microcosms were spiked at 50 ng L^{-1}.

Table S4. Estrogen LC-ESI(–)-MS/MS transitions.

Analyte	RT[a,b] (min)	Precursor (*m/z*)	Product (*m/z*)	Q = Quant ion
E2-*d4*	4.71	275	147	Q
		275	187	
E2	4.76	271	145	
		271	183	Q
E1	5.20	269	143	
		269	145	Q
monoBrE2	6.06	349	79	
		351	81	Q
diClE2	7.31	339	307	Q
		341	309	
diBrE2	7.94	429	79	
		429	81	Q
diBrE1[c]	8.22	429	81	Q

[a] RT: retention time

[b] Phenyl-hexyl column (Agilent Poroshell 2.1 × 50 mm; 2.7 µm)

[c] The quantitation transitions used for diBrE1 and diBrE2 were identical (429/81). For diBrE2, this transition represents the $[M\text{-}H]^-$ ion for the $^{79}Br^{81}Br$ combination. For diBrE1, which is 2 Da lighter, the same transition represents $[M\text{-}H]^-$ for the $^{81}Br^{81}Br$ combination. Baseline separation of diBrE1 and diBrE2 allowed us to quantify both dibromo derivatives.

Table S5. Summary of abiotic model fit parameters for river microcosms spiked with free and halogenated estrogens. Uncertainties were calculated by non-linear regression analysis (MATLAB; *fitnlm*) and represent ±1 standard error.

**Experiment**	Estrogen	Microcosm Bottle	Initial Conditions	Lumped Abiotic Rate Constant (k_a) (h^{-1})[a]	Abiotic Reaction Order (n)
**BD-1805**					
	E2	A,B,C	50 ng L^{-1}; RW; sediment	0.058 ± 0.026	2.7 ± 0.8
	E1	A,B,C	50 ng L^{-1}; RW; sediment	na	na
**BD-1810**					
	E2	A,B,C	50 ng L^{-1}; RW; sediment	0.024 ± 0.004	1.31 ± 0.24
	E1	A,B,C	50 ng L^{-1}; RW; sediment	na	na
**BD-1906**					
	E2	E	50 ng L^{-1}; RW	na	na
	monoBrE2	E	50 ng L^{-1}; RW	na	na
	diBrE2	E	50 ng L^{-1}; RW	na	na
	diClE2	E	50 ng L^{-1}; RW	na	na
	E1	E	50 ng L^{-1}; RW	na	na
	diBrE1	E	50 ng L^{-1}; RW	na	na
	E2	A	50 ng L^{-1}; RW	na	na
	E1	A	50 ng L^{-1}; RW	na	na
	E2	B	50 ng L^{-1}; RW	na	na
	E1	B	50 ng L^{-1}; RW	na	na
	E1	F	50 ng L^{-1}; RW	na	na
	E1	S	50 ng L^{-1}; RW; sediment	na	na
**BD-1907**					
	E2	ESH	1250 ng L^{-1}; RW; sediment	0.0193 ± 0.0005	1.38 ± 0.05
	monoBrE2	ESH	1250 ng L^{-1}; RW; sediment	0.053 ± 0.006	1.44 ± 0.13
	diBrE2	ESH	1250 ng L^{-1}; RW; sediment	0.27 ± 0.07	2.54 ± 0.27
	diClE2	ESH	1250 ng L^{-1}; RW; sediment	0.19 ± 0.06	2.3 ± 0.3
	E1	ESH	1250 ng L^{-1}; RW; sediment	na	na

diBrE1	ESH	1250 ng L^{-1}; RW; sediment	na	na
E2	ESL	50 ng L^{-1}; RW; sediment	0.016 ± 0.003	1.4 ± 0.4
monoBrE2	ESL	50 ng L^{-1}; RW; sediment	0.067 ± 0.006	1.70 ± 0.11
diBrE2	ESL	50 ng L^{-1}; RW; sediment	0.061 ± 0.023	1.8 ± 0.4
diClE2	ESL	50 ng L^{-1}; RW; sediment	nd	nd
E1	ESL	50 ng L^{-1}; RW; sediment	na	na
diBrE1	ESL	50 ng L^{-1}; RW; sediment	na	na
E2	EH	1250 ng L^{-1}; RW	na	na
monoBrE2	EH	1250 ng L^{-1}; RW	na	na
diBrE2	EH	1250 ng L^{-1}; RW	na	na
diClE2	EH	1250 ng L^{-1}; RW	na	na
E1	EH	1250 ng L^{-1}; RW	na	na
diBrE1	EH	1250 ng L^{-1}; RW	na	na
E2	EL	50 ng L^{-1}; RW	na	na
monoBrE2	EL	50 ng L^{-1}; RW	na	na
diBrE2	EL	50 ng L^{-1}; RW	na	na
diClE2	EL	50 ng L^{-1}; RW	na	na
E1	EL	50 ng L^{-1}; RW	na	na
diBrE1	EL	50 ng L^{-1}; RW	na	na

[a] The lumped abiotic rate constant (k_a) and its corresponding unit (h^{-1}) assumes that data are plotted as R_t/R_0 vs. time. Note that direct comparisons of k_a values are confounded by the influence of different n and R_0 values.

na = Not applicable.

nd = Not determined due to low abundance peak areas.

Figure S1. Estrogen structures, chemical names, and abbreviations used in the present study.

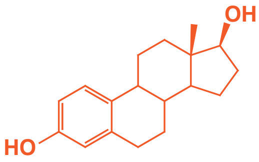

17β-estradiol
(E2)

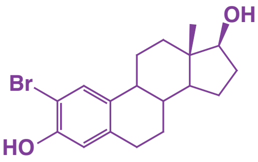

2-bromo-17β-estradiol
(monoBrE2)

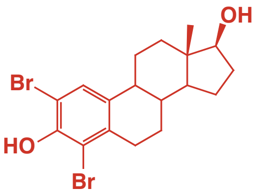

2,4-dibromo-17β-estradiol
(diBrE2)

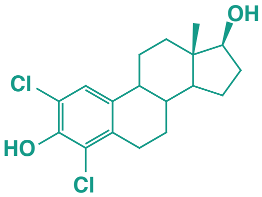

2,4-dichloro-17β-estradiol
(diClE2)

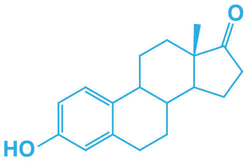

estrone
(E1)

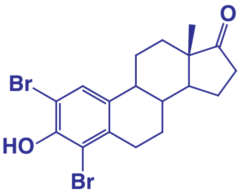

2,4-dibromo-estrone
(diBrE1)

Figure S2. Representative estrogen (E2) plots from BD-1907 illustrate the modeling approach used in this study. Modeled fits, differential equations, and analytical solutions are shown for the river water-sediment microcosms (top panel; filled symbols) and the river water-only microcosms (bottom panel; open symbols). Derivations and additional details are presented in the SI section titled "Modeling Approach and Derivations."

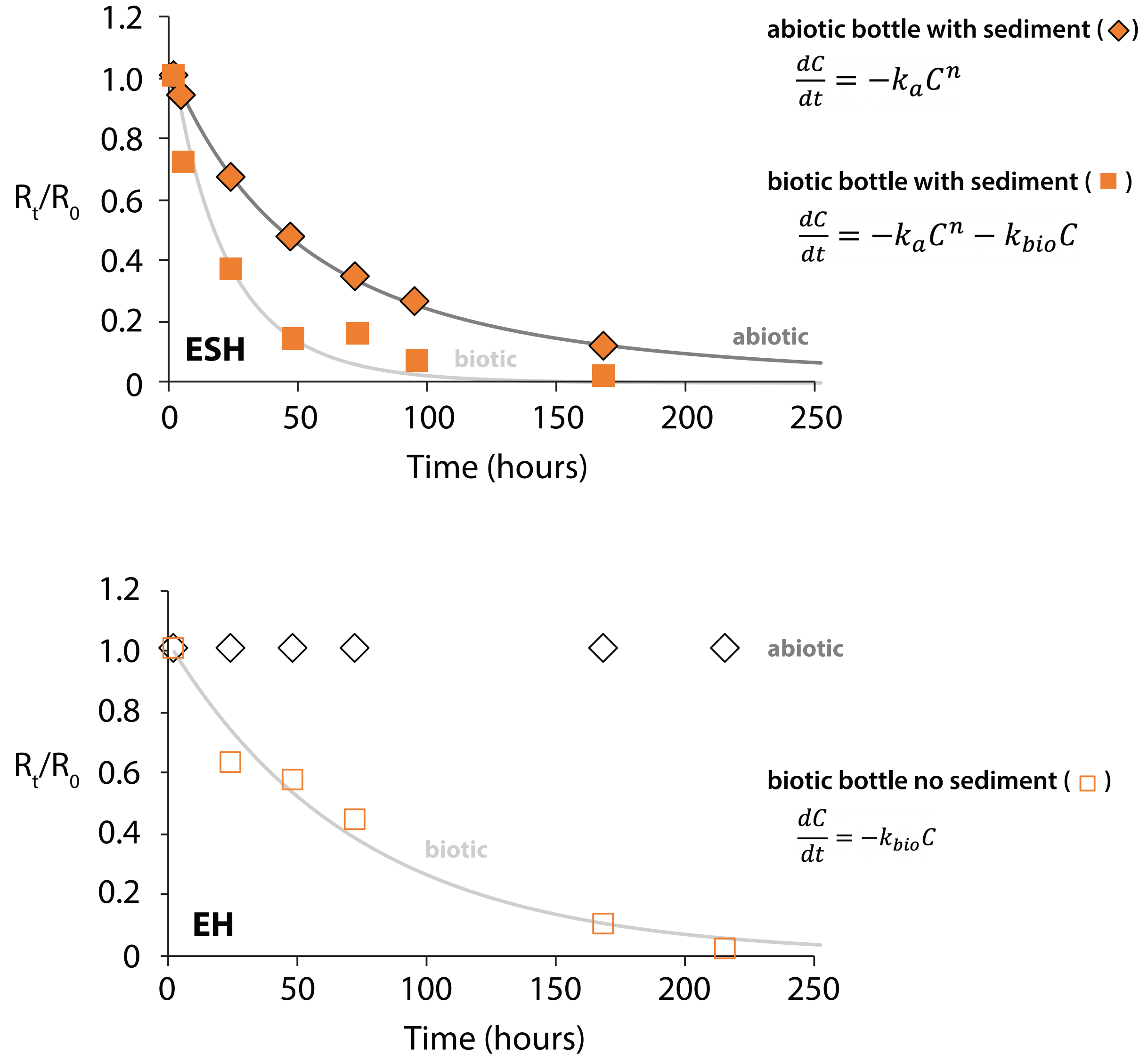

Figure S3. Estrogen degradation in river water microcosms (BD-1906). All data are normalized to the internal standard, time zero, and the corresponding abiotic (diamonds) microcosm. Modeled fits assume first order kinetics as described in the main text and detailed in the SI. The top four panels represent data acquired from a single river water microcosm spiked with a mixture of E2 (orange), monoBrE2 (purple), diBrE2 (red), and diClE2 (green). The bottom left panel shows E2 biodegradation kinetics from three replicate bottles (one of which was a mixture) and the bottom right panel shows how E1 biodegradation differs in river water-only and water-sediment microcosms.

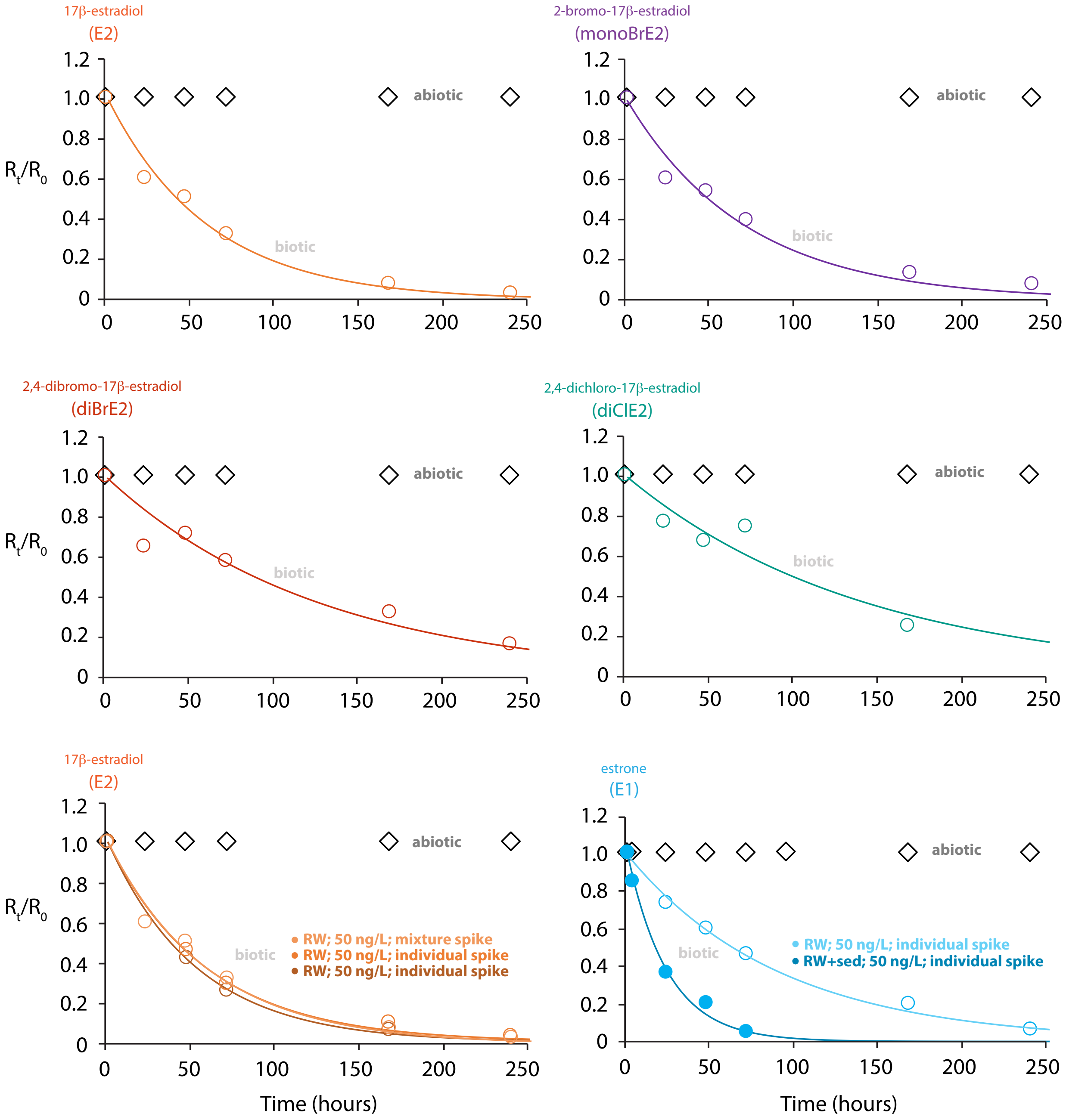

Figure S4. Estrogen degradation in BD-1907 river water microcosms with (ESH, ESL; filled symbols) and without (EH, EL; open symbols) sediment, at high (ESH, EH; squares) and low (ESL, EL; circles) spike concentrations, and under biotic and abiotic (diamonds) conditions. Modeled fits were determined according to analytical solutions to differential equations described in the main text and detailed in the SI. Panels are labeled with the corresponding estrogen and color-coded: E2 (orange), monoBrE2 (purple), diBrE2 (red), diClE2 (green).

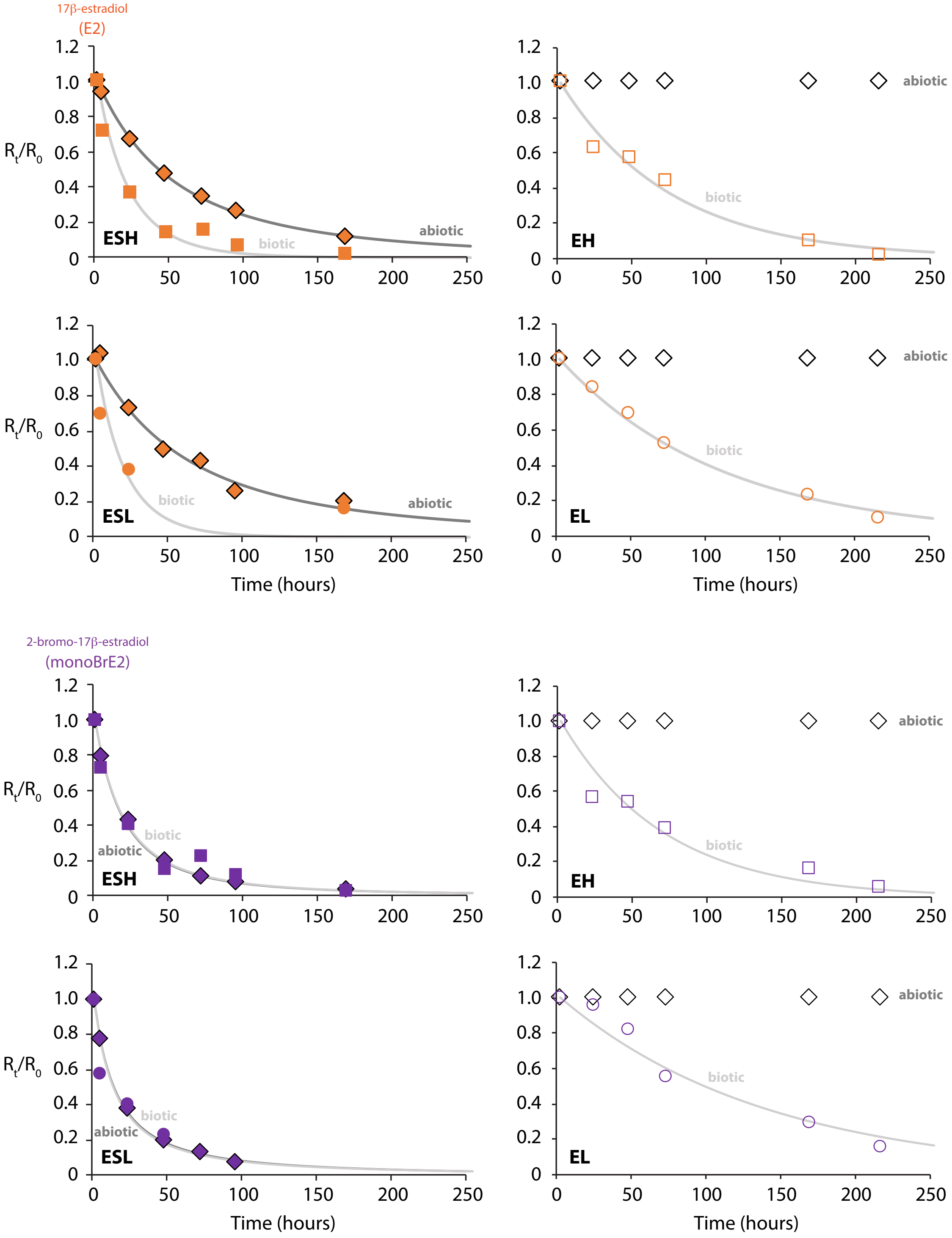

2,4-dibromo-17β-estradiol
(diBrE2)

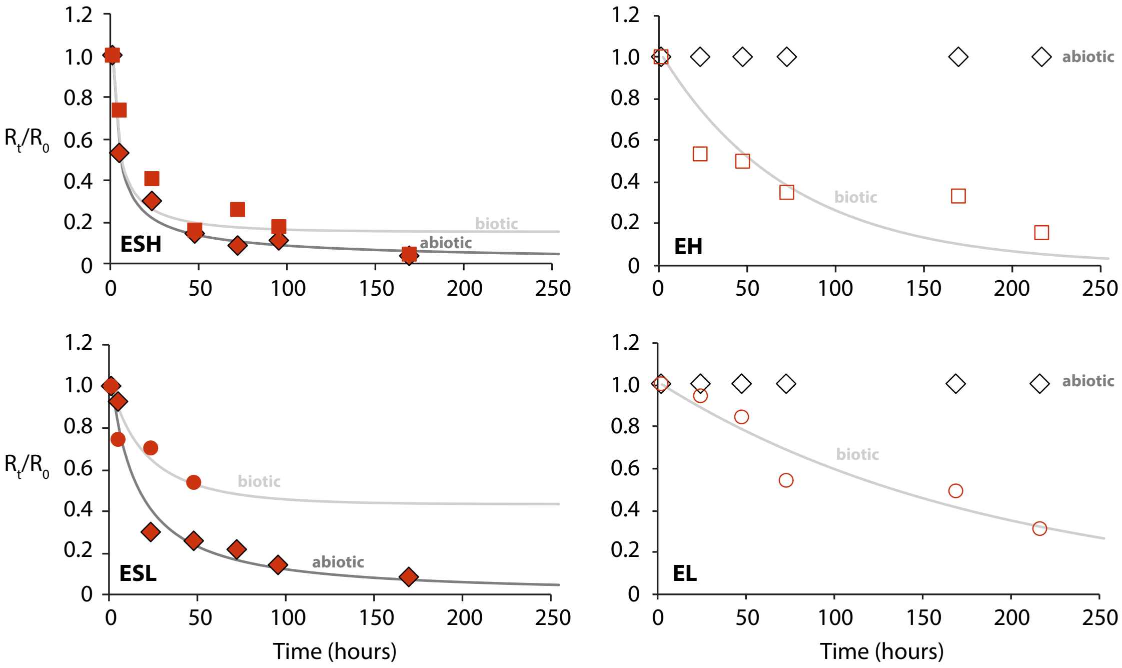

2,4-dichloro-17β-estradiol
(diClE2)

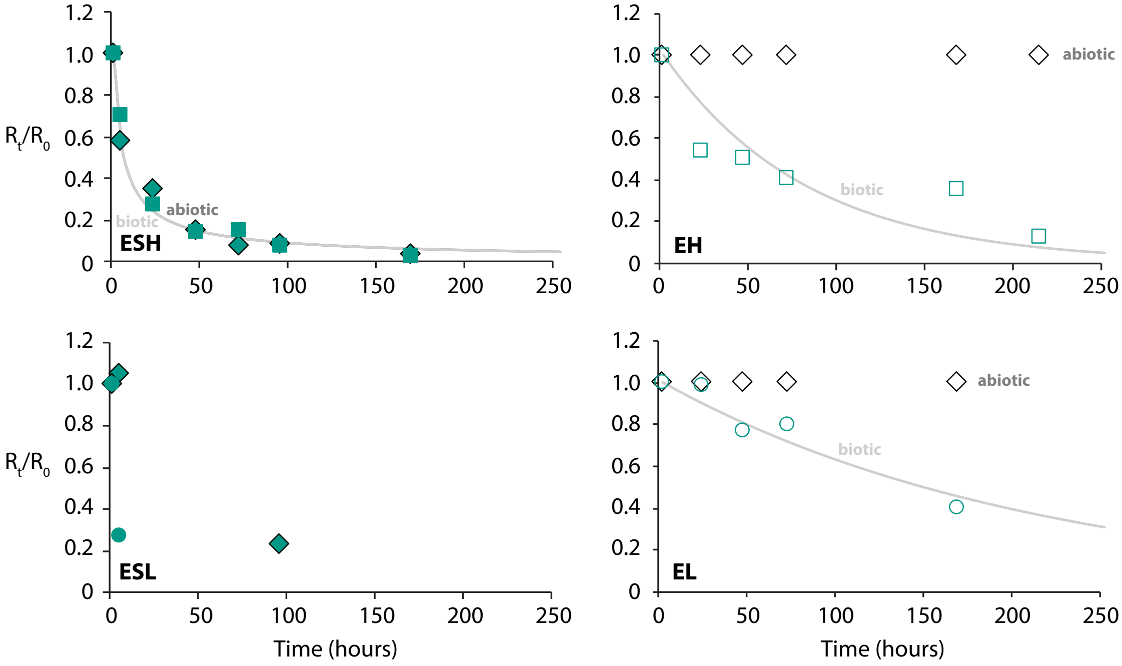

Figure S5. Microcosm ATP, DO, and pH trends for BD-1906. Microcosm labels (e.g., F, A, B) and characteristics are detailed in Table S3.

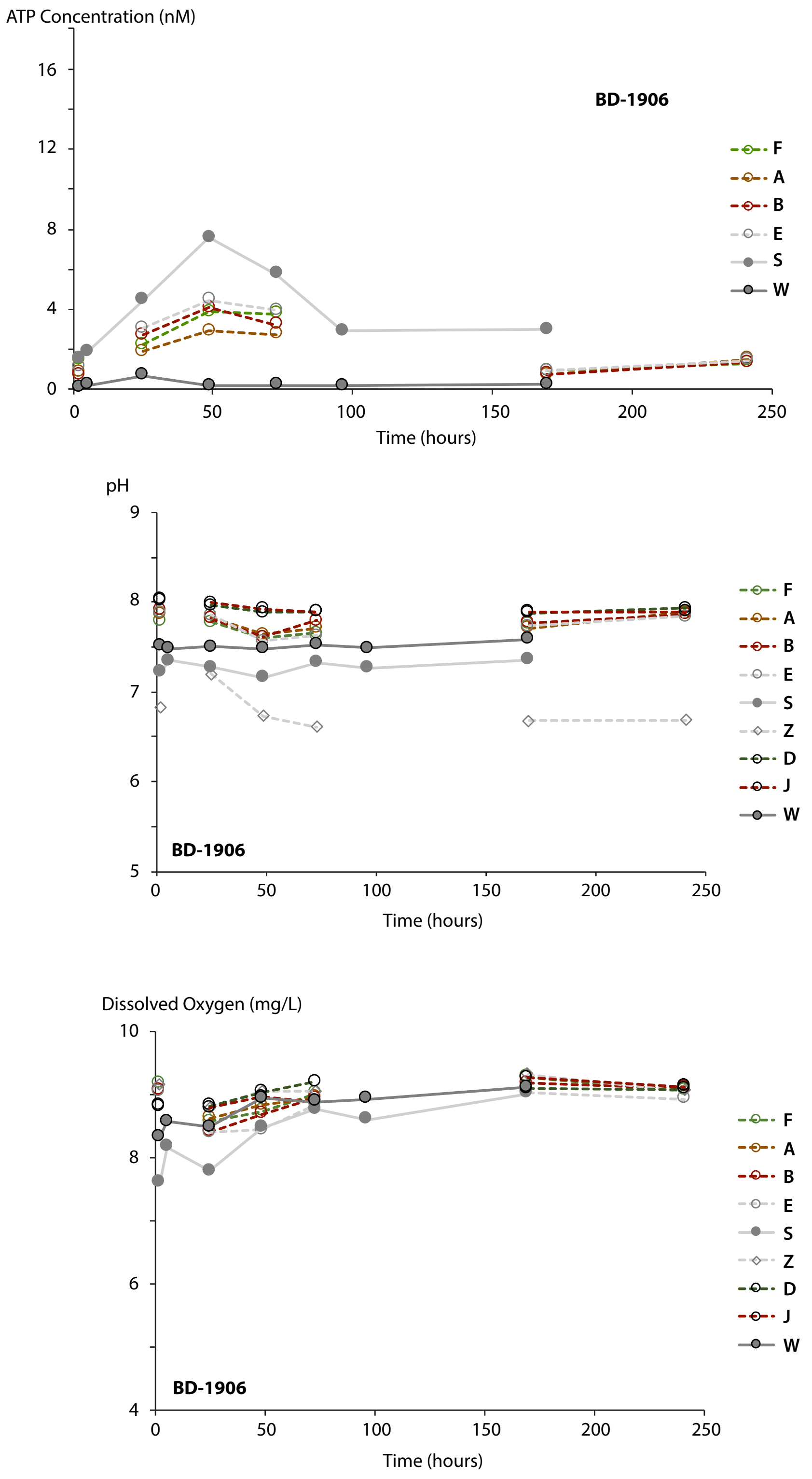

Figure S6. Microcosm ATP, DO, and pH trends for BD-1907. Microcosm labels (e.g., EL, ESL, EH) and characteristics are detailed in Table S3.

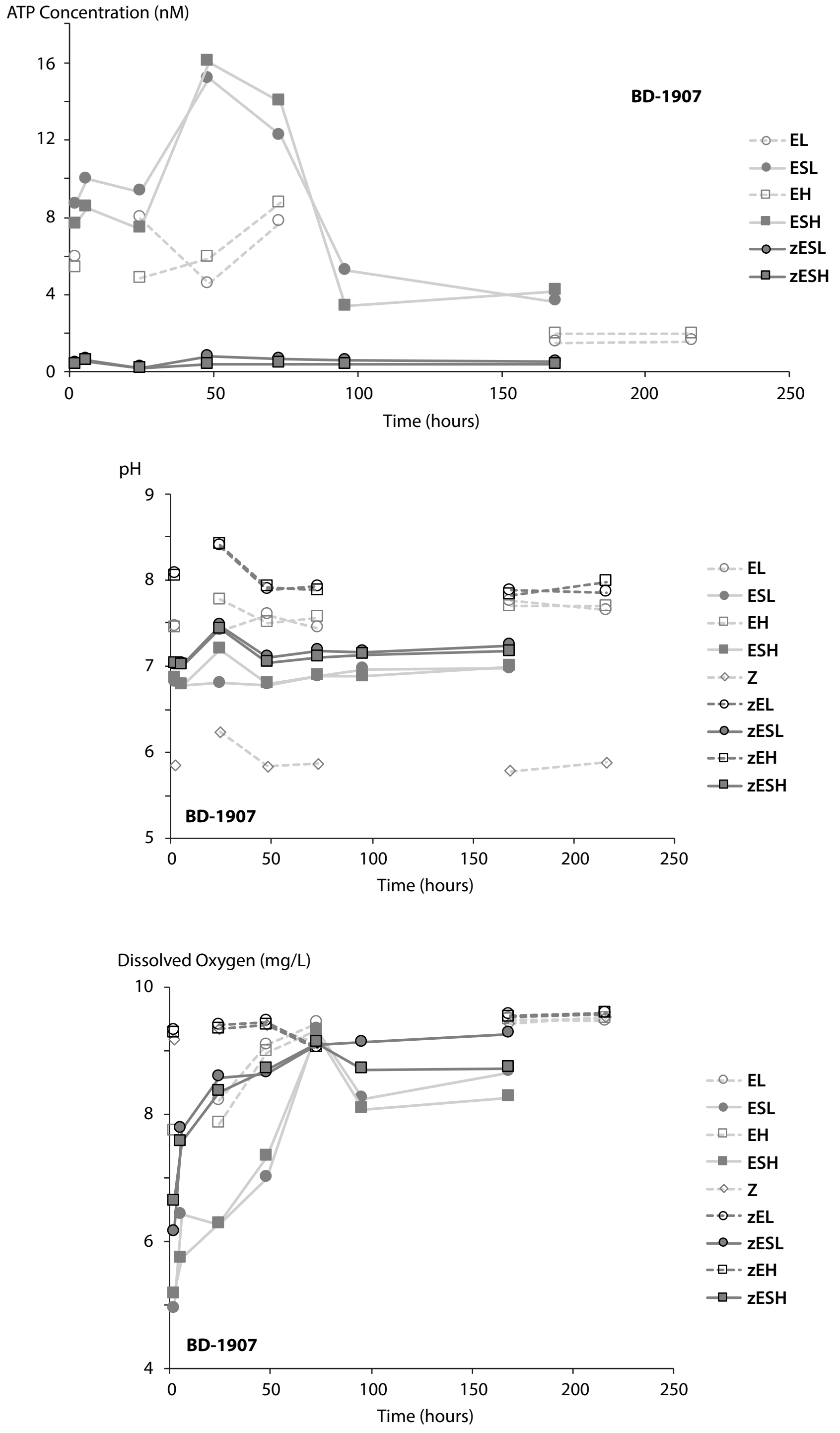

Figure S7. Nitrogen-based nutrient trends during BD-1907 for microcosms with sediment (ESH, ESL; filled symbols) and without sediment (EH, EL; open symbols) spiked with a mixture of E2, monoBrE2, diBrE2, and diClE2 at 1250 ng L^{-1} (ESH, EH; squares) and 50 ng L^{-1} (ESL, EL; circles).

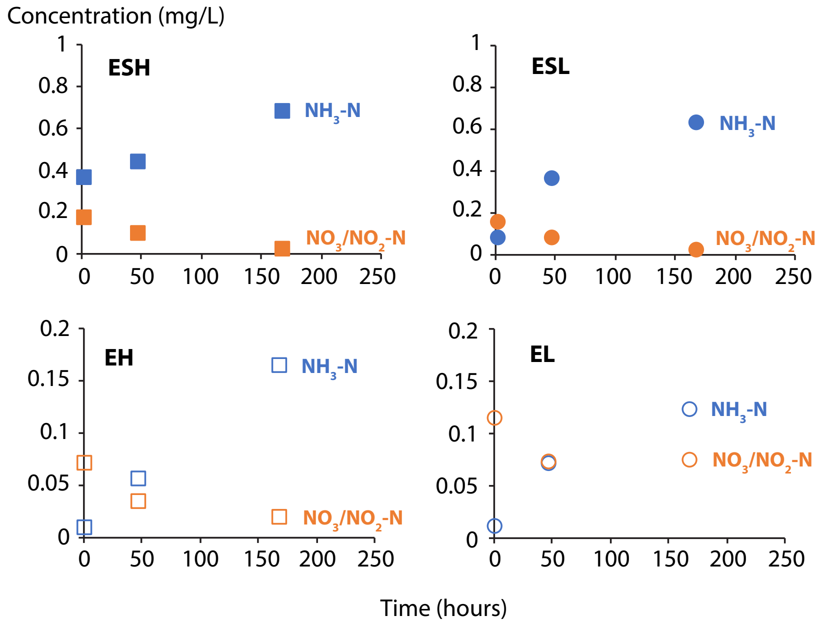

Figure S8. Microbial communities identified using 16S rRNA gene sequencing during the BD-1810 experiment. Relative abundance at the class level is shown for in-situ river water ("R"), time zero ("t0"; 1.6 h), time three ("t3"; 48.8 h), and time six ("t6"; 169 h) in each of three pools: sediment, 0.22 µm filters, and 3 µm filters.

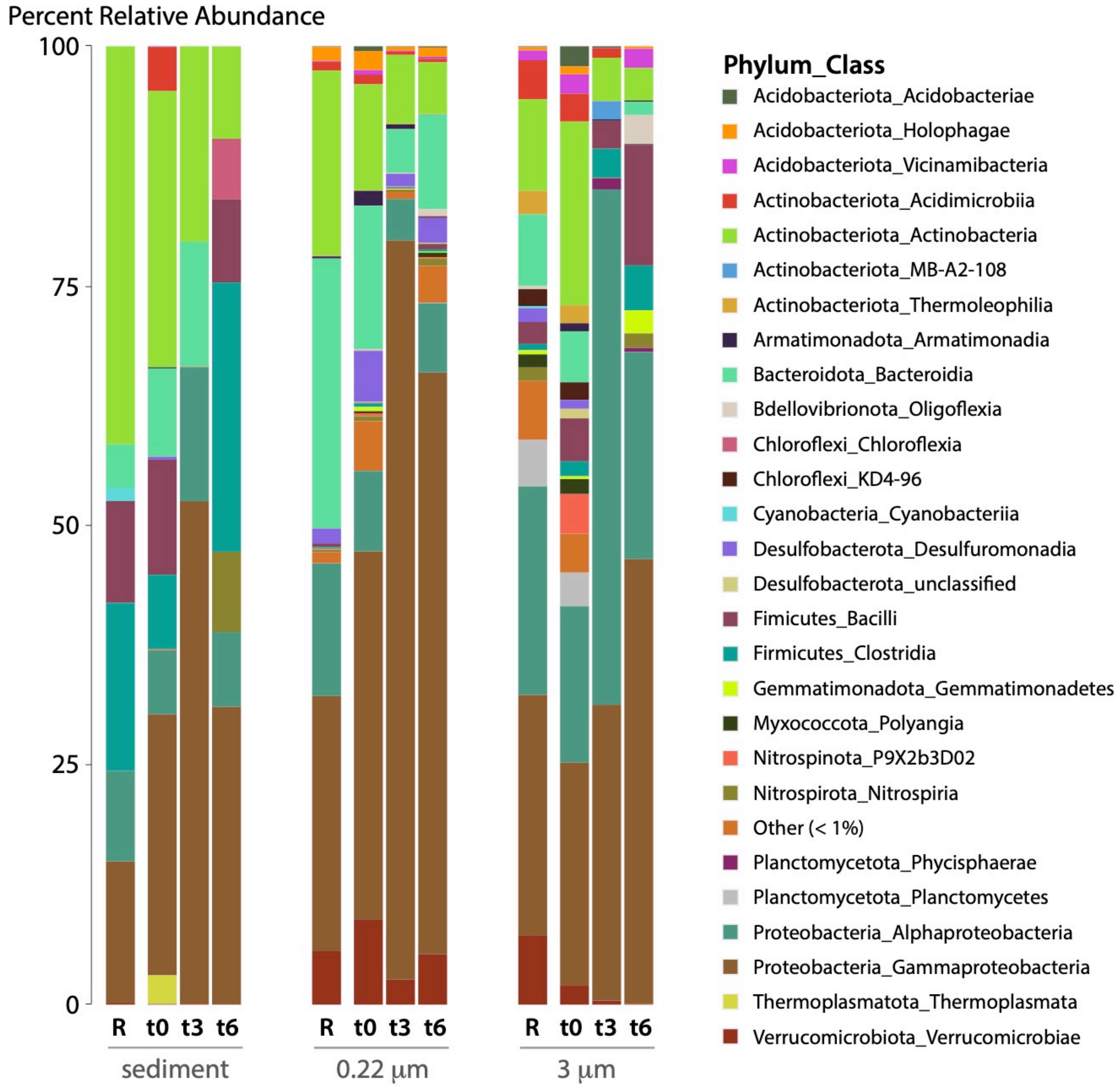

Figure S9. Microbial communities identified using 16S rRNA gene sequencing for the BD-1810 experiment. Relative abundance at the genus level is shown for in-situ river water ("River"), time zero ("t0"; 1.6 h), time three ("t3"; 48.8 h), and time six ("t6"; 169 h) in each of three pools: sediment, 0.22 µm filters, and 3 µm filters. Only those organisms with non-zero abundance in at least three of the four samples (River, t0, t3, t6) for at least one of the three pools (sediment, 0.22 µm, 3 µm) are shown.

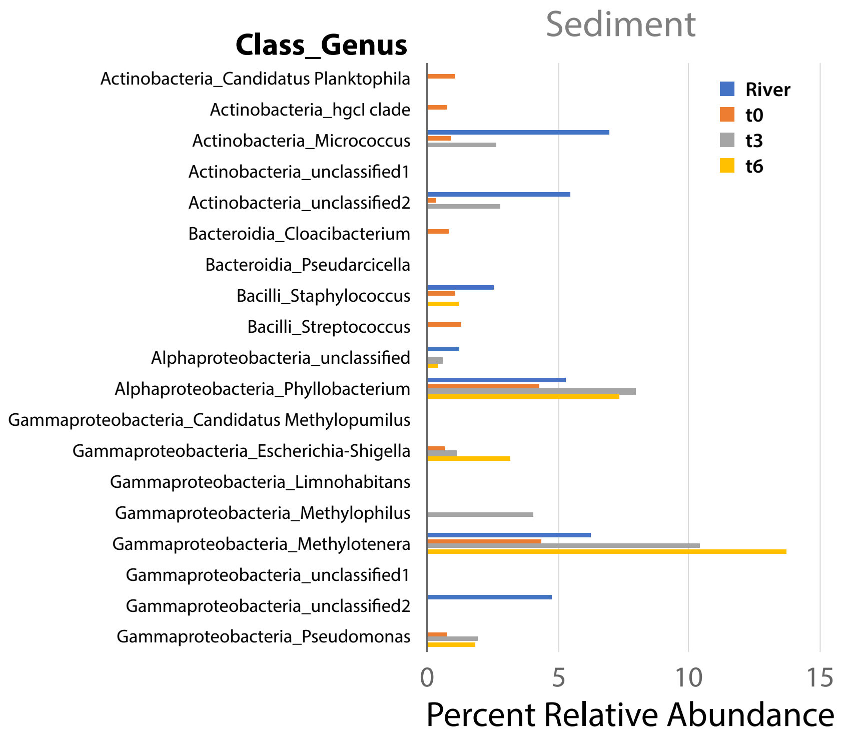

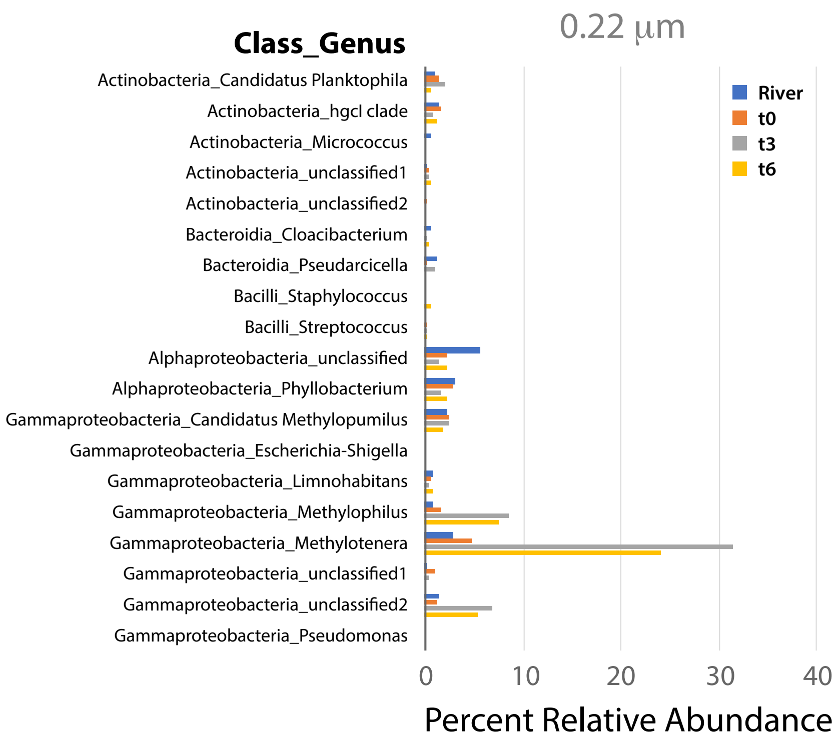

0.22 µm
Class_Genus
Actinobacteria_Candidatus Planktophila
Actinobacteria_hgcI clade
Actinobacteria_Micrococcus
Actinobacteria_unclassified1
Actinobacteria_unclassified2
Bacteroidia_Cloacibacterium
Bacteroidia_Pseudarcicella
Bacilli_Staphylococcus
Bacilli_Streptococcus
Alphaproteobacteria_unclassified
Alphaproteobacteria_Phyllobacterium
Gammaproteobacteria_Candidatus Methylopumilus
Gammaproteobacteria_Escherichia-Shigella
Gammaproteobacteria_Limnohabitans
Gammaproteobacteria_Methylophilus
Gammaproteobacteria_Methylotenera
Gammaproteobacteria_unclassified1
Gammaproteobacteria_unclassified2
Gammaproteobacteria_Pseudomonas
River
t0
t3
t6
0
10
20
30
40
Percent Relative Abundance

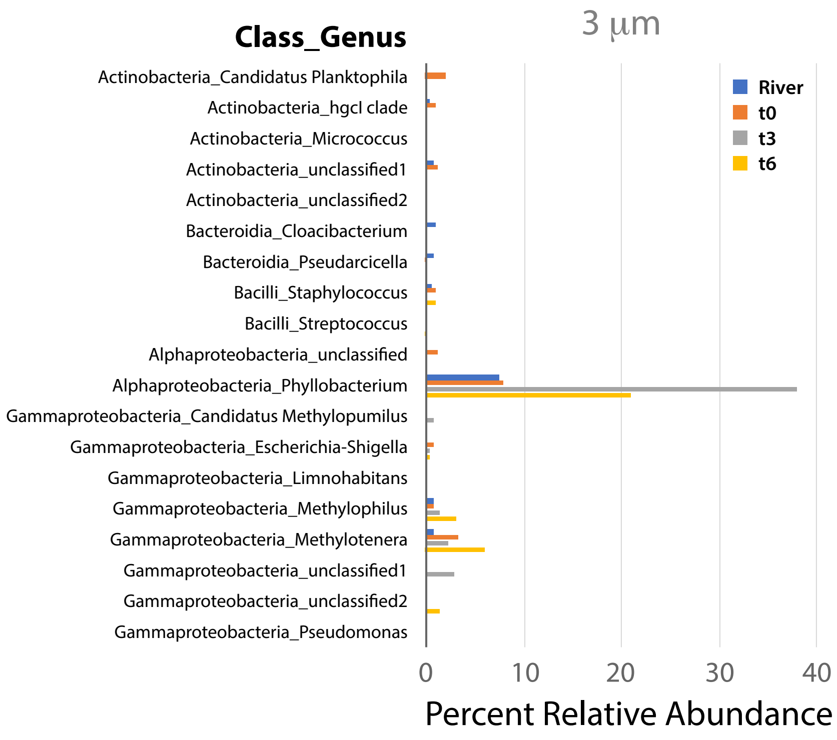

3 µm
Class_Genus
Actinobacteria_Candidatus Planktophila
Actinobacteria_hgcI clade
Actinobacteria_Micrococcus
Actinobacteria_unclassified1
Actinobacteria_unclassified2
Bacteroidia_Cloacibacterium
Bacteroidia_Pseudarcicella
Bacilli_Staphylococcus
Bacilli_Streptococcus
Alphaproteobacteria_unclassified
Alphaproteobacteria_Phyllobacterium
Gammaproteobacteria_Candidatus Methylopumilus
Gammaproteobacteria_Escherichia-Shigella
Gammaproteobacteria_Limnohabitans
Gammaproteobacteria_Methylophilus
Gammaproteobacteria_Methylotenera
Gammaproteobacteria_unclassified1
Gammaproteobacteria_unclassified2
Gammaproteobacteria_Pseudomonas
River
t0
t3
t6
0
10
20
30
40
Percent Relative Abundance

Figure S10. Small scale microcosm experiments point to sediment (Sed) as having had the greatest effect on ATP concentrations, while methanol (MeOH; co-solvent) and 17β-estradiol (E2; spike) did not impact ATP dynamics significantly in river water (RW) microcosms. Water was replenished at 524 h by removing 100 mL and replacing it with a new 100 mL. Only bottles that initially received MeOH and E2 showed increases in ATP after a respike event at 656 h, suggesting that, over time, the microbial community may acclimate to utilizing MeOH and E2. Samples labeled "E2" contain methanol as the co-solvent. Ultrapure water is indicated by the abbreviation "DI".

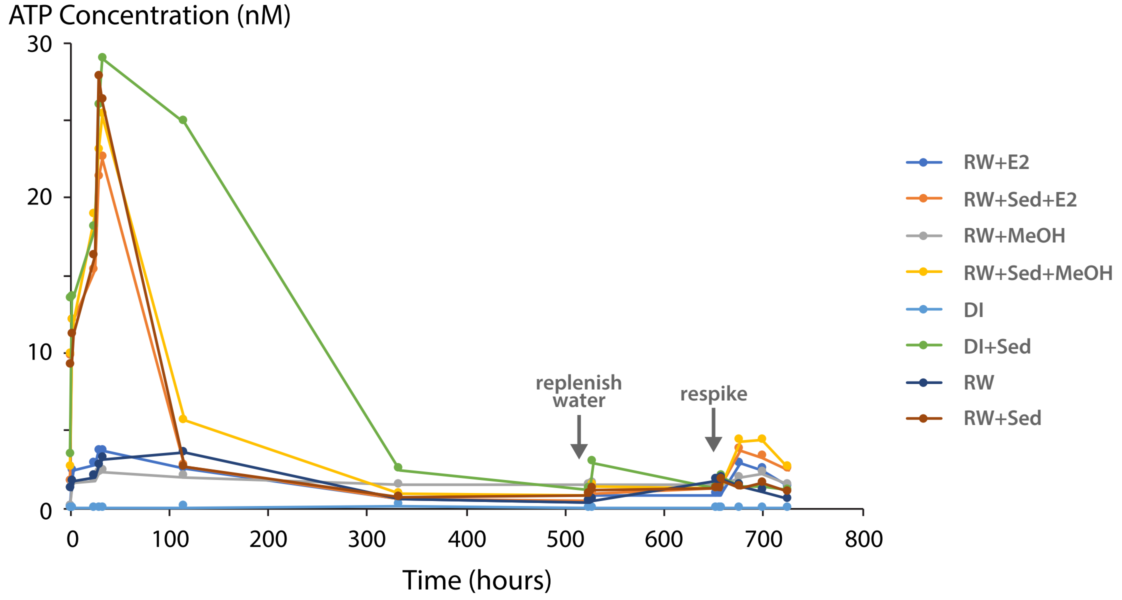

Figure S11. Small scale microcosm experiments using different combinations of the co-solvents methanol (MeOH), acetonitrile (ACN), and dimethyl sulfoxide (DMSO) and river water (RW) upstream (U) and downstream (D) from a wastewater treatment plant outfall (Willow Lake WWTP, Salem, OR) indicated that upstream and downstream water had similar ATP trends, that MeOH and DMSO enhance ATP levels slightly relative to RW, and that ACN spikes (with and without E2) resulted in the smallest deviations relative to RW-only microcosms.

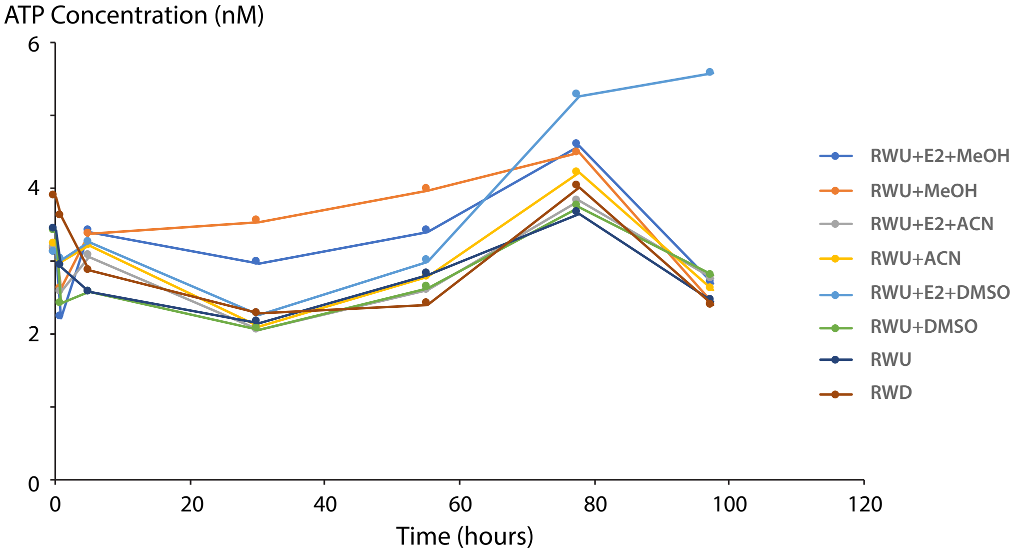

Figure S12. Representative ATP calibration curve used during BD-1907. The intercept was determined to be statistically indistinguishable from zero and so the regression line (slope = 26,213 nM^{-1}; $R^2 = 0.99999$) was forced through the origin.

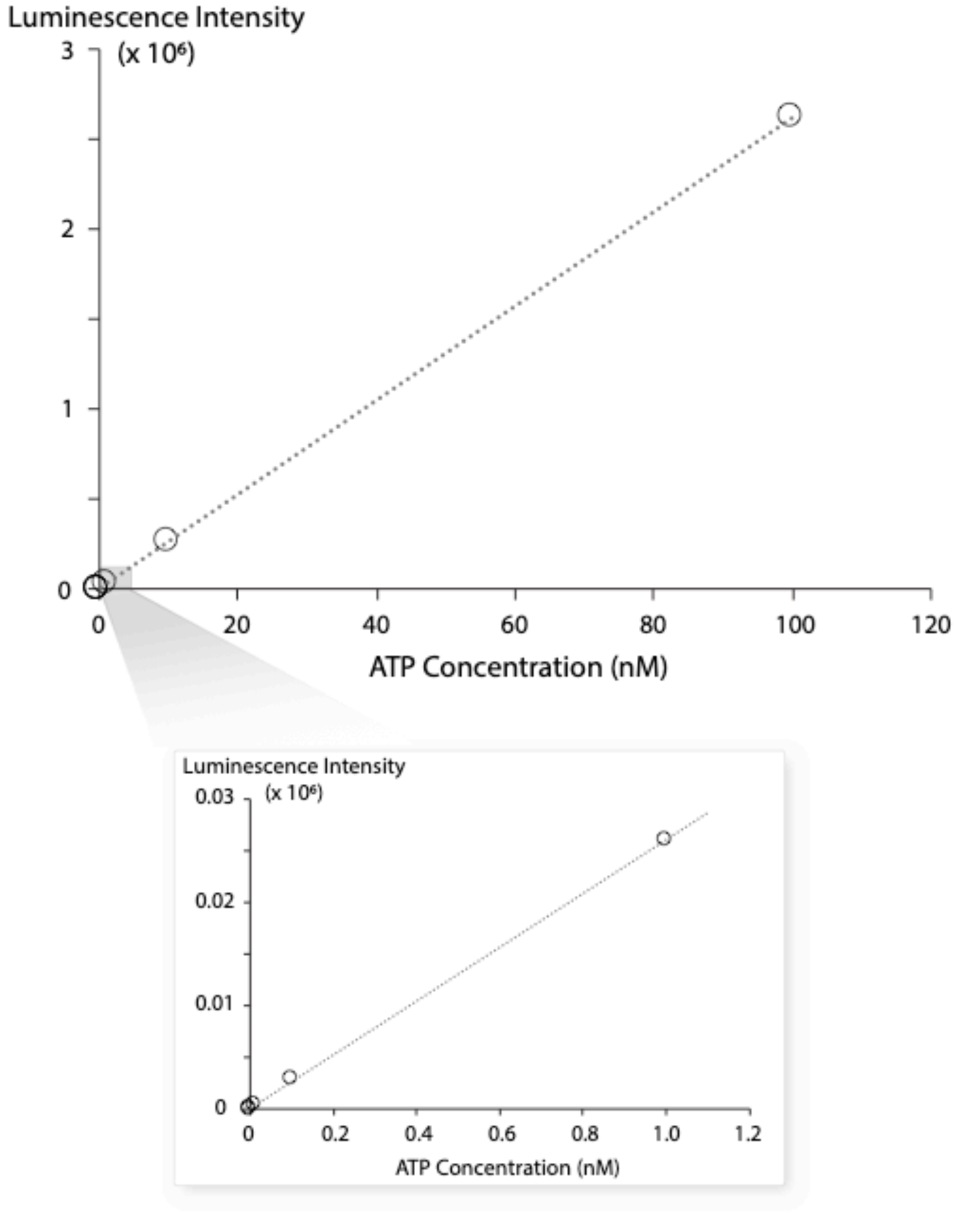

Ancillary Methods

Ancillary measurements were made at selected time points during each experiment using water removed from microcosms after estrogen samples were taken. These measurements included temperature, pH, specific UV absorbance ($SUVA_{254}$), 16S rRNA, as well as the concentration of dissolved oxygen, ATP, dissolved organic carbon (DOC), nutrients (ammonium, nitrate/nitrite, phosphate, and sulfate), anions (chloride, bromide, and fluoride), and total suspended solids (TSS).

Temperature, pH, and dissolved oxygen

Temperature, pH, and dissolved oxygen were measured immediately after ancillary water was removed from each microcosm. The pH electrode (ROSS; Thermo-Fisher Scientific) and optical dissolved oxygen/temperature probe (EcoSense ODO200; YSI) were calibrated daily.

Nutrients, anions, dissolved organic carbon, $SUVA_{254}$, and total suspended solids

Nutrient and anion samples (~30 mL) were syringe-filtered using a 0.22 μm cartridge filter (Sterivex) into acid-washed polycarbonate bottles, stored at -20 °C, and analyzed in batches at the Oregon State University Cooperative Chemical Analytical Laboratory (OSU-CCAL) according to standard methods (https://ccal.oregonstate.edu/methodology). Dissolved organic carbon (DOC) and $SUVA_{254}$ samples (~40 mL) were filtered using a Nucleopore polycarbonate membrane filter (0.4 μm; Whatman) previously rinsed with ultrapure water and housed in a stainless-steel filter holder. This filter was chosen for its demonstrated low DOC leaching.[8] DOC samples were collected in acid-washed polycarbonate bottles, stored at -20 °C, and measured at OSU-CCAL using a Shimadzu TOC-VCSH combustion analyzer. Filtered $SUVA_{254}$ samples were collected in baked (450 °C; 5 h) 4 mL amber vials. Absorbance measurements were made in matched quartz cuvettes (1 cm; Starna Cells) on a dual-beam UV-vis spectrophotometer (Agilent Cary 300) and blank-corrected. $SUVA_{254}$ was calculated as the absorbance of a sample at 254 nm normalized to the dissolved organic carbon concentration (Equation X).

$$\mathrm{SUVA}_{254}\ (\mathrm{L\ mg\ C^{-1}\ m^{-1}}) = \frac{Abs\ at\ 254\ nm}{DOC\ (mg\ L^{-1})} \qquad (0)$$

Total suspended solids were measured on 0.45 μm filters (47 mm; GF/F; Whatman) that had been prepared via successive rounds of ultrapure water rinsing and baking at 450 °C (5 h). Samples were filtered using a 3-port vacuum manifold (< 20 mmHg) with glass reservoirs. Filters were then baked for 30 minutes at 100°C and placed in a desiccator for 24 hours before TSS mass was calculated by difference.

ATP

As a proxy for microbial activity, ATP was quantitated using the BacTiter-Glo assay (Promega) where the luminescence signal was measured using a microplate reader (Infinite M200 Pro; Tecan). After a five-minute incubation at 38 °C, 50 μL of thawed BacTiter-Glo reagent was added to one row of a white 96-well plate while 250 μL of sampled water was added to a separate row

of the same plate. The plate was incubated for one minute at 38 °C. The water was then transferred to the wells containing BacTiter-Glo using an Eppendorf multichannel pipettor. Luminescence was measured as a raw signal and converted to a concentration (nM) using a standard ATP calibration curve generated for each batch (e.g., Figure S12). BacTiter-Glo reagent was prepared via the manufacturer's protocol with a rest period of approximately 2.5 hours to "burn off" ATP and increase sensitivity. Aliquots were prepared in appropriate volumes for each time point and stored at -80°C.

ATP calibration curves were prepared for each new batch of BacTiter-Glo from a 10,000 nM stock solution (Promega) and used to correct for slight variability in assay performance across experiments. Previous work[9] found a good correlation between ATP concentrations and microbial cellular abundance. Standards were made in filtered and autoclaved ultrapure water (0.10 μm, 121°C, 30 minutes) and stored in a -20°C freezer until use. The procedure used to build the calibration curve followed the same protocols as described above and all signals were corrected using ultrapure water controls.

16S rRNA gene sequence analysis

The microbial community was characterized using the 16S rRNA gene from various environmental samples in the Willamette River during the October 2018 microcosm experiment (BD-1810). Water samples from the river and microcosm were collected in 0.22 μm Sterivex cartridges (EMD Millipore; Burlington, MA) and 3 μm Nucleopore filters (47 mm; Whatman; Marlborough, MA). Sediment samples were also used for analysis.

Willamette River sediment samples were collected using sterile techniques from the same site (45º 00.53' N, 123º 04.25' W) used in the microcosm experiments. Sediments were stored in WhirlPaks, placed on dry ice during transport to the laboratory, and stored at -80°C. Willamette River water was collected nearby (45º 00.52' N, 123º 04.30' W) and filtered on site using a portable peristaltic pump and acid-washed PharMed BPT tubing (Saint-Gobain Life Sciences; Akron, OH) connected in series through a 3 μm filter housed in an acid-washed polycarbonate filter holder (47 mm) followed by a 0.22 μm cartridge filter. Each filter was then placed in a WhirlPak, transported to the laboratory on dry ice, and stored at -80°C.

Sediment and water were collected from a replicate microcosm prepared and spiked exactly like those used for estrogen kinetics measurements for the specific purpose of genetic analysis. Approximately 400 mL of microcosm water was collected using a solvent-rinsed 100 mL volumetric pipette at each of three timepoints after the initial estrogen spike (1.6 h, 48.8 h, and 168.8 h). This water was then pumped through the 3 μm and 0.22 μm filters, and stored according to the procedure described above. Microcosm sediment was collected using a sterilized cocktail spoon and stored at in a sterile polyethylene tube at -80°C.

Sediment and 3 µm filter extractions

Research Fecal/Soil Microbe Microprep kit (Zymo Research; Irvine, CA) was used to extract DNA for all sediment and 3 µm filter samples at designated time points. Samples were extracted in batches of 2 – 4 samples at a time alongside a control. The control for sediment samples consisted of following the procedure using only the reagents in the kit. The control for the 3 µm filter was a sterile filter.

Sediment extraction utilized approximately 500 mg of the sample. The 3 µm filter sample was cut into pieces using a sterile stainless-steel surgical blade. Each sample was processed in the Fisher Scientific Bead Mill 24. Successive rounds of centrifugation with the addition of a lysis buffer and preparation buffers allowed for the cells to lyse and release the DNA into the sample. DNA was eluted using nuclease free water and stored in a refrigerator at approximately 35°F until PCR.

Sterivex extractions

The DNeasy PowerWater Sterivex kit (Qiagen; Germantown, MD) was used to extract DNA from all Sterivex samples at each time point. Samples were extracted in batches of 2 – 4 samples at a time alongside a control. The control was a sterile sterivex filter. All water was removed from the cartridge either during sampling or directly before extraction using a 60 mL syringe. The DNA was eluted using nuclease free water and stored in a refrigerator at approximately 35°F until PCR.

Polymerase chain reaction (PCR)

16S rRNA gene PCR was used to amplify samples from BD-1810 samples. PCR cleanup was performed using the EZ-10 Spin Column PCR Products Purification Kit BS364. Cleaned PCR products were stored in the refrigerator until further use, or in the freezer until sequencing.

Gel electrophoresis

Agarose gels (Fisher BioReagents Agarose, diluted 1X TAE from Thermo Scientific 50X TAE Electrophoresis Buffer) were prepared to visualize the amplified DNA product using ethidium bromide as the nucleic acid stain. Gels were run on the PowerPac Basic at 100 V for 35 minutes. Gels were visualized using the BioRad ChemiDoc XRS+ Universal Hood II.

Amplicon sequences analysis

The amplicon sequence variants (ASV) tables were generated using the DADA2 pipeline (v 1.16). Reads were demultiplexed and chimeras were removed, which were subsequently utilized for the creation of the ASV taxonomy and counts table. The Silva database version 138.1 reference was employed to build the ASV taxonomy table and assign sequence variants to their appropriate taxonomic categories. The removal of contaminants was performed using the “decontam” package from DADA2. ASV tables were then exported and used to calculate relative abundance of groups of interest within the experimental samples.

Ancillary Results

Dissolved organic carbon (DOC)

River DOC concentrations (1.5 – 3.3 mg L^{-1}) matched those measured in microcosms (1.2 – 3.4 mg L^{-1}) with one exception (BD-1805) in which elevated values (2.3 – 14.1 mg L^{-1}) were observed in the microcosms. On average, the concentration of DOC decreased by 51% over the course of each experiment.

Nutrients

Microcosm nutrient levels mirrored those found in river water, where chloride, fluoride, and bromide concentrations were approximately 3 mg L^{-1}, 0.02 mg L^{-1}, and <0.01 mg L^{-1}, respectively. Initial phosphate levels in microcosms never exceeded 0.022 mg L^{-1} and changed little over time. Sulfate concentrations averaged 1 mg L^{-1}.

Sorption Estimates

The extent to which free and halogenated estrogens sorb to microcosm sediments was estimated by assuming a two-phase system at equilibrium and calculating the fraction of each estrogen in the sediment phase (f_{is}) and the water phase (f_{iw}) according to the equation below.

$$f_{is} = 1 - f_{iw} = 1 - \frac{1}{1 + \left[\left(\frac{1}{1 + 10^{pH - pK_a}} \right) K_{oc}^{HA} f_{oc} r_{sw} \right]}$$

Parameter descriptions:

	Description
Microcosm	
r_{sw} (kg_{solid}/L_w)	Ratio of solid to water in a microcosm
$f_{oc,\ low}$ (kg_{oc}/kg_{solid})	Fraction organic carbon in microcosm sediments (low estimate)
$f_{oc,\ high}$ (kg_{oc}/kg_{solid})	Fraction organic carbon in microcosm sediments (high estimate)
pH	Average pH of all sediment-containing microcosms
Estrogen	
pK_a	Acid dissociation constant (see Table S1)
K_{oc}^{HA} (L_w/L_{oc})	Organic carbon-water distribution coefficient for the protonated form (see Table S1); We assume that log K_{oc} ~ log K_{oc}^{HA}
Calculated	
$f_{is,\ low}$	Fraction of estrogen (i) in sediment phase (calculated using $f_{oc,\ low}$)
$f_{is,\ high}$	Fraction of estrogen (i) in sediment phase (calculated using $f_{oc,\ high}$)

Parameter inputs and sorption estimates:

Microcosm		BD-1805	BD-1810	BD-1906	BD-1907	AVG
	r_{sw} (kg_{solid}/L_w)	0.0157	0.0205	0.0147	0.0116	
	$f_{oc,\ low}$ (kg_{oc}/kg_{solid})[a]	0.002	0.002	0.002	0.002	
	$f_{oc,\ high}$ (kg_{oc}/kg_{solid})[b]	0.01	0.01	0.01	0.01	
	pH	7.062	7.369	7.395	7.018	
**E1**				**E1**		
pK_a = 10.77	$f_{is,\ low}$	0.05	0.06	0.04	0.03	**0.05**
log K_{oc} (L_w/L_{oc})[a] = 3.19	$f_{is,\ high}$	0.20	0.24	0.19	0.15	**0.19**
**E2**				**E2**		
pK_a = 10.71	$f_{is,\ low}$	0.05	0.07	0.05	0.04	**0.05**
log K_{oc} (L_w/L_{oc})[b] = 3.24	$f_{is,\ high}$	0.21	0.26	0.20	0.17	**0.21**
**monoBrE2**				**monoBrE2**		
pK_a = 8.99	$f_{is,\ low}$	0.06	0.07	0.05	0.04	**0.06**
log K_{oc} (L_w/L_{oc})[b] = 3.28	$f_{is,\ high}$	0.23	0.28	0.21	0.18	**0.22**
**diBrE2**				**diBrE2**		
pK_a = 7.50	$f_{is,\ low}$	0.18	0.19	0.14	0.15	**0.16**
log K_{oc} (L_w/L_{oc})[b] = 3.99	$f_{is,\ high}$	0.53	0.54	0.45	0.46	**0.49**
**diClE2**				**diClE2**		
pK_a = 7.43	$f_{is,\ low}$	0.18	0.18	0.13	0.14	**0.16**
log K_{oc} (L_w/L_{oc})[b,c] = 3.99	$f_{is,\ high}$	0.52	0.52	0.43	0.45	**0.48**

[a] (Lee et al 2003)[6]

[b] (Casey et al 2017)[7]

[c] diClE2 was assumed to have the same log K_{oc} value as diBrE2

Note: "low" estimates assumed f_{oc} = 0.2%; "high" estimates assumed f_{oc} = 1%

Photolysis

Photolysis kinetics in the Willamette River

To facilitate comparisons between biodegradation and photolysis in the Willamette River (Figure 2), we determined photolysis rate constants for E2, monoBrE2, diBrE2, and diClE2 at 1 mg L^{-1} initial concentration in river water and ultrapure water at pH 7.6 during May-June 2018 under natural solar irradiance using previously described methods.[10] Photolysis quantum yields, half-lives, and rate constants are reported below.

Estrogen	Exposure Date	Observed Half-life ($t_{1/2,obs}$) (min)	Observed Rate Constant (k_{obs}) (min^{-1})	Quantum Yield (mol Ein^{-1})
**Ultrapure Water**				
E2	21 May - 14 Jun 2018	$(1.85 \pm 0.20) \times 10^{4}$	$(3.7 \pm 0.4) \times 10^{-5}$	$(1.95 \pm 0.22) \times 10^{-2}$
monoBrE2	21 May 2018	188 ± 7	$(3.69 \pm 0.14) \times 10^{-3}$	$(2.38 \pm 0.17) \times 10^{-1}$
diBrE2	21 May 2018	16.3 ± 0.2	$(4.25 \pm 0.06) \times 10^{-2}$	$(1.78 \pm 0.07) \times 10^{-1}$
diClE2	21 May 2018	44.5 ± 0.8	$(1.558 \pm 0.027) \times 10^{-2}$	$(1.98 \pm 0.23) \times 10^{-1}$
**Willamette River Water (0.45μm filtered)**				
E2	21 May - 14 Jun 2018	$(8.70 \pm 0.21) \times 10^{3}$	$(7.97 \pm 0.19) \times 10^{-5}$	-
monoBrE2	21 May 2018	80.0 ± 2.4	$(8.66 \pm 0.26) \times 10^{-3}$	-
diBrE2	21 May 2018	14.4 ± 0.4	$(4.83 \pm 0.15) \times 10^{-2}$	-
diClE2	21 May 2018	30.2 ± 0.4	$(2.29 \pm 0.03) \times 10^{-2}$	-

E2: 17β-estradiol
monoBrE2: 2-bromo-17β-estradiol
diBrE2: 2,4-dibromo-17β-estradiol
diClE2: 2,4-dichloro-17β-estradiol

Concentration dependence

Separate experiments were conducted to characterize the sensitivity of diBrE2 photolysis to initial concentrations between 50 ng L^{-1} – 1 mg L^{-1} in both ultrapure water and Suwannee River Humic Acid (5 mg L^{-1}) at pH 7.0 under natural solar irradiance following previously described methods.[10] Photolysis rate constants and half-lives are reported below.

Estrogen	Exposure Date	Initial Concentration	Observed Half-life ($t_{1/2,obs}$) (min)	Observed Rate Constant (k_{obs}) (min^{-1})
**Ultrapure Water**				
diBrE2[a]	26 Jul 2017	1 mg L^{-1}	58.3 ± 0.9	$(1.189 \pm 0.019) \times 10^{-2}$
diBrE2[b]	26 Jul 2017	10 μg L^{-1}	43.1 ± 1.5	$(1.61 \pm 0.06) \times 10^{-2}$
diBrE2[c]	26 Jul 2017	50 ng L^{-1}	47 ± 8	$(1.48 \pm 0.25) \times 10^{-2}$
**Suwannee River Humic Acid (5 mg L^{-1})**				
diBrE2[a]	26 Jul 2017	1 mg L^{-1}	42.0 ± 0.6	$(1.650 \pm 0.025) \times 10^{-2}$
diBrE2[b]	26 Jul 2017	10 μg L^{-1}	60 ± 4	$(1.16 \pm 0.08) \times 10^{-2}$
diBrE2[c]	26 Jul 2017	50 ng L^{-1}	86 ± 15	$(8.03 \pm 1.4) \times 10^{-3}$

diBrE2: 2,4-dibromo-17β-estradiol
[a] Quantitation by LC-UV at 290 nm
[b] Quantitation by LC-MS/MS (*m/z* 429 > 81) without internal standard
[c] Quantitation by LC-MS/MS (*m/z* 429 > 81) with internal standard (E2-*d4*; *m/z* 275 > 147)

Photolysis rate constant corrections under field conditions

To minimize bias during photolysis studies we employ natural solar irradiance, filtered whole water, actinometers, dark controls, and no co-solvents. Despite these efforts, it is likely that environmental photolysis rates are slower due to several factors such as tube geometry, light screening in natural waters, and 24-hour averaging. Considered together, these corrections (described below) suggest that the photolysis of free and halogenated estrogens in the Willamette River may be 10 – 40 times slower than in tube experiments.

Tube geometry correction:[11]

$$k_{p,tot}^{0} = k_{obs}/1.5$$

where k_{obs} is the observed photolysis rate constant determined using tube experiments and $k_{p,tot}^{0}$ is the near surface photolysis rate constant

Light screening correction:[12]

$$k_{p,tot} = S(\lambda) k_{p,tot}^{0}$$

where $k_{p,tot}$ is the depth-averaged photolysis rate constant and $S(\lambda)$ is the light screening factor, defined as

$$S(\lambda) \cong \frac{1 - 10^{-(1.2)z\alpha(\lambda_m)}}{2.303(1.2)z\alpha(\lambda_m)}$$

where z is the vertical water depth through which screening is occurring, λ_m is the wavelength at which the specific rate of light absorption is at a maximum, and $\alpha(\lambda_m)$ is the absorbance through a 1 cm pathlength of river water at λ_m

24-hour correction:[12]

$$k_{p,tot(24h)} \cong 0.4 k_{p,tot(noon)}$$

where $k_{p,tot(24h)}$ is the 24-hour averaged depth-averaged photolysis rate constant and $k_{p,tot(noon)}$ is the depth-averaged photolysis rate constant at noon.

Field-corrected kinetic parameters for estrogen photolysis in the Willamette River ($z \sim 200$ cm) are reported below along with biodegradation half-lives from river water microcosms (BD-1906). The use of field-corrected photolysis rates does not change the fact that, in the Willamette River, halogenated estrogen photolysis is much faster than biodegradation, but for free estrogens photolysis is significantly slower than biodegradation.

	E2	monoBrE2	diBrE2	diClE2
Observed Photolysis Rate Constant, k_{obs} (min^{-1})	7.97×10^{-5}	8.66×10^{-3}	4.83×10^{-2}	2.29×10^{-2}
Observed Photolysis Half-Life, $t_{1/2,obs}$ (min)	8700	80.0	14.4	30.2
Near Surface Photolysis Rate Constant, $k_{p,tot}^0$ (min^{-1})	5.31×10^{-5}	5.77×10^{-3}	3.22×10^{-2}	1.53×10^{-2}
Wavelength of Maximum Specific Light Absorption Rate, λ_m (nm)	347	311	317	311
Beam Attenuation Coefficient at the Wavelength of Maximum Specific Light Absorption Rate, $\alpha(\lambda_m)$ (cm^{-1})	0.0107	0.0175	0.0162	0.0175
Light Screening Factor, $S(\lambda)$ (assuming z = 200 cm)	0.169	0.103	0.111	0.103
Depth-Averaged Photolysis Rate Constant, $k_{p,tot}$ (min^{-1})	8.98×10^{-6}	5.96×10^{-4}	3.59×10^{-3}	1.58×10^{-3}
24h-Averaged, Depth-Averaged Photolysis Rate Constant, $k_{p,tot(24h)}$ (min^{-1})	8.98×10^{-6} [a]	2.39×10^{-4} [b]	1.43×10^{-3} [b]	6.32×10^{-4} [b]
24h-Averaged, Depth-Averaged Photolysis Half-Life, $t_{1/2,p,tot(24h)}$ (min)	77200	2910	480	1100
Biodegradation (BD-1906) Half-Life, $t_{1/2,bio}$ (min)	2490	2940	5280	5880

E2: 17β-estradiol
monoBrE2: 2-bromo-17β-estradiol
diBrE2: 2,4-dibromo-17β-estradiol
diClE2: 2,4-dichloro-17β-estradiol
[a] E2 photolysis data were collected over several weeks (21 May – 14 June, 2018) so we do not apply a 24h correction
[b] Halogenated E2 photolysis data were collected between 1pm – 5pm on 21 May, 2018; we apply the full noon-to-24h correction

Modeling Approach and Derivations

Modeling estrogen kinetics in abiotic and biotic microcosms

Estrogen peak areas were converted to internal standard (E2-*d4*) normalized peak area ratios (R_t) according to

$$R_t = \frac{Estrogen\ Peak\ Area}{Internal\ Standard\ Peak\ Area} \tag{1}$$

To facilitate comparisons between different experiments and estrogens, peak area ratios (R_t) were further normalized to the peak area ratio at time zero (R_0) and plotted as R_t/R_0, which served as a proxy for estrogen concentration. For those bottles containing only river water, corresponding azide (NaN_3) control data were used to correct for any abiotic processes at work. The resulting data were fit in MATLAB (*fitnlm*) according to pseudo-first order kinetics

$$C_t = C_0 e^{-k_{bio}t} \tag{2}$$

where k_{bio} is the biodegradation rate constant and C_t and C_0 are concentrations at time t and time zero, respectively. Our expectation that estrogen biodegradation would exhibit first order behavior was supported by a range of theoretical and empirical studies[6, 13-17] as well as data from our own river water microcosms (Figure S3 and Figure S4).

Sediment-containing bottles

In the presence of sediment, estrogen data did not follow simple first order kinetics and significant removals were observed in abiotic bottles. Others have observed similar behavior in the presence of sediments, soils, and sludge.[18-20] Since sorption and other abiotic processes are relevant in microcosms containing sediment, a modeling approach that could extract biodegradation rate constants from the more complex river water-sediment microcosm data was required. A variety of methods have been used to "subtract" abiotic processes in order to isolate biodegradation rate constants in similar systems.[13, 20] Our approach was conceptually similar to multiexponential curve fitting,[21] curve peeling,[22] or successive subtraction,[23] and it involved solving paired biotic and abiotic differential equations using non-linear regression analysis (MATLAB; *fitnlm*). In short, we modeled abiotic removals (sorption, redox) using a general n^{th} order term and treated biodegradation as a pseudo-first order process as shown by

$$\frac{dC}{dt} = -k_a C^n - k_{bio} C \tag{3}$$

where C represents concentration and k_{a} and k_{bio} are the lumped abiotic and biodegradation rate constants, respectively. For abiotic microcosms, Equation 3 simplifies to

$$\frac{dC}{dt} = -k_a C^n \tag{4}$$

which yields the following analytical solution

$$C_t = \left(-k_a(1-n)t + C_0^{(1-n)}\right)^{\frac{1}{(1-n)}} \quad (5)$$

where n is the reaction order, t is time in hours and C_0 is the initial concentration. Since we use R_t/R_0 as our proxy for concentration, by definition

$$C_0 = \left(\frac{R_t}{R_0}\right)_0 = \left(\frac{R_0}{R_0}\right) = 1 \quad (6)$$

Kinetic data from each abiotic water-sediment microcosm were fit according to Equation 5, which was solved by non-linear regression (MATLAB; *fitnlm*), yielding parameter estimates for k_a and n. The fit values of n averaged 1.9 (ranging from 1.3 – 2.7; Table S5). These estimates are reasonable given the fact that sorption is a concatenation of several complex processes, and sorption kinetics generally simplify to pseudo-first or pseudo-second order kinetics under a variety of theoretical treatments.[24-26] Concurrent abiotic oxidation reactions such as those catalyzed by minerals or natural organic matter would also be included in this general n^{th} order abiotic term. The parameters estimated from abiotic data (k_a and n) were then applied to the biotic microcosm model described below since abiotic processes are expected to occur at similar rates in biotic and abiotic systems.

Since both abiotic and biotic processes are at work in biotic microcosms containing sediment, these data were fit using the following analytical solution to Equation 3:

$$C_t = \left(\frac{k_{bio}e^{\varphi}}{1-k_a e^{\varphi}}\right)^{\frac{1}{(n-1)}} \quad (7)$$

where

$$\varphi = -k_{bio}(n-1)t + ln\left(\frac{C_0{}^{(n-1)}}{k_{bio}+k_a C_0{}^{(n-1)}}\right) \quad (8)$$

The expanded version of Equation 7 is therefore

$$C_t = \left(\frac{k_{bio}e^{(-k_{bio}(n-1)t+ln\left(\frac{C_0{}^{(n-1)}}{k_{bio}+k_a C_0{}^{(n-1)}}\right))}}{1-k_a e^{(-k_{bio}(n-1)t+ln\left(\frac{C_0{}^{(n-1)}}{k_{bio}+k_a C_0{}^{(n-1)}}\right))}}\right)^{\frac{1}{(n-1)}} \quad (9)$$

The biotic river water-sediment microcosm data were fit using MATLAB (*fitnlm*) according to Equation 9 after substituting k_a and n from the abiotic fit (Equation 5), yielding an estimate for the pseudo-first order biodegradation rate constant (k_{bio}). Uncertainties for all fit parameters were

calculated in MATLAB by the same non-linear model function (*fitnlm*) and represent ±1 standard error.

Product formation and decay kinetics

In abiotic microcosms containing the parent estrogens E2 and diBrE2, we observed growth of the oxidized estrone derivatives E1 and diBrE1. The processes responsible for this growth may include oxidation reactions or biodegradation by azide-resistant microbes. In abiotic microcosms, growth of daughter estrogens (E1 and diBrE1) was modeled according to

$$\frac{dC_d}{dt} = k_a C_p \quad (10)$$

where C_p *and* C_d represent the concentration of the parent (e.g., E2) and daughter (e.g., E1), respectively, and k_a is the rate constant for the abiotic processes fit using the daughter data. In biotic microcosms, where formation and degradation of daughter estrogens by biodegradation are also at work, the equation becomes

$$\frac{dC_d}{dt} = (k_a + k_{bio,p})C_p - k_{bio,d}C_d \quad (11)$$

where $k_{bio,p}$ and $k_{bio,d}$ are the biodegradation rate constants of the parent (e.g., E2) and daughter (e.g., E1), respectively. Solving Equation 10 and 11 for C_d yields

$$C_d = C_{p,0}(1 - e^{-k_a t}) + C_{d,0} \quad (12)$$

and

$$C_d = \left(\frac{k_a + k_{bio,p}}{k_{bio,d} - (k_a + k_{bio,p})}\right) C_{p,0}\left(e^{-(k_a + k_{bio,p})t} - e^{-k_{bio,d}t}\right) + C_{d,0}\left(e^{-k_{bio,d}t}\right) \quad (13)$$

where the initial concentrations of the daughter ($C_{d,0}$) and parent ($C_{p,0}$) were known, the biodegradation rate constant of the parent ($k_{bio,p}$) came from the parent estrogen data (Equation 9; k_{bio}), and the value of k_a was determined by fitting the abiotic data (Equation 10). The biodegradation rate constant of the daughter ($k_{bio,d}$) was then estimated according to Equation 13.

Derivation of modeling equations

The analytical solution for the general n^{th} order equation was derived in the following steps:

$$\frac{dC}{dt} = -k_a C^n$$

$$\int_{C_0}^{C_t} C^{-n}\, dC = \int_0^t -k_a dt$$

$$\frac{C_t^{(1-n)}}{1-n} - \frac{C_0^{(1-n)}}{1-n} = -k_a t$$

$$C_t^{(1-n)} - C_0^{(1-n)} = -k_a(1-n)t$$

$$C_t^{(1-n)} = -k_a(1-n)t + C_0^{(1-n)}$$

$$C_t = \left(-k_a(1-n)t + C_0^{(1-n)}\right)^{\frac{1}{(1-n)}}$$

The analytical solution for the two-term equation used to model the biotic river water-sediment data was derived in the following steps:

$$\frac{dC}{dt} = -k_a C^n - k_{bio} C$$

$$\frac{dC}{k_a C^n + k_{bio} C} = -dt$$

$$\int_{C_0}^{C_t} \frac{dC}{k_a C^n + k_{bio} C} = \int_0^t -dt$$

$$\int_{C_0}^{C_t} \frac{dC}{C(k_{bio} + k_a C^{(n-1)})} = -t$$

After making the substitutions

$m = n - 1$

$$a = k_a$$
$$b = k_{bio}$$

we use integral 84 from the CRC Handbook of Chemistry and Physics (63rd edition, p. A-36),

$$\int \frac{dx}{x(b + ax^m)} = \frac{1}{bm} \ln\left(\frac{x^m}{b + ax^m}\right)$$

to obtain

$$\frac{1}{bm} \ln\left(\frac{C_t^m}{b + aC_t^m}\right) - \frac{1}{bm} \ln\left(\frac{C_0^m}{b + aC_0^m}\right) = -t$$

$$\ln\left(\frac{C_t^m}{b + aC_t^m}\right) - \ln\left(\frac{C_0^m}{b + aC_0^m}\right) = -bmt$$

The following substitution,

$$\theta = \left(\frac{C_0^m}{b + aC_0^m}\right)$$

results in

$$\ln\left(\frac{C_t^m}{b + aC_t^m}\right) = -bmt + \ln(\theta)$$

$$\frac{C_t^m}{b + aC_t^m} = e^{-bmt + \ln(\theta)}$$

$$\frac{1}{\frac{b}{C_t^m} + a} = e^{-bmt + \ln(\theta)}$$

An additional substitution,

$$\varphi = -bmt + \ln(\theta)$$

gives

$$\frac{1}{\frac{b}{C_t^m} + a} = e^{\varphi}$$

$$1 = e^{\varphi}\left(\frac{b}{C_t^m} + a\right)$$

$$1 = \left(\frac{be^{\varphi}}{C_t^m} + ae^{\varphi}\right)$$

$$1 - ae^{\varphi} = \frac{be^{\varphi}}{C_t^m}$$

$$\frac{1}{1 - ae^{\varphi}} = \frac{C_t^m}{be^{\varphi}}$$

$$\frac{e^{\varphi}}{1 - ae^{\varphi}} = \frac{C_t^m}{b}$$

$$C_t^m = \frac{be^{\varphi}}{1 - ae^{\varphi}}$$

$$C_t = \left(\frac{be^{\varphi}}{1 - ae^{\varphi}}\right)^{\frac{1}{m}}$$

After reversing the simplifying substitutions above, we get

$$C_t = \left(\frac{k_{bio}e^{\varphi}}{1 - k_a e^{\varphi}}\right)^{\frac{1}{(n-1)}}$$

and the fully expanded analytical solution,

$$C_t = \left(\frac{k_{bio}e^{(-k_{bio}(n-1)t + ln\left(\frac{C_0{}^{(n-1)}}{k_{bio} + k_a C_0{}^{(n-1)}}\right))}}{1 - k_a e^{(-k_{bio}(n-1)t + ln\left(\frac{C_0{}^{(n-1)}}{k_{bio} + k_a C_0{}^{(n-1)}}\right))}}\right)^{\frac{1}{(n-1)}}$$

References

1. USEPA *Estimation Programs Interface Suite for Microsoft Windows, v 4.11*; Washington, DC, USA, 2013.

2. Hilal, S. H.; Karickhoff, S. W.; Carreira, L. A. *Prediction of Chemical Reactivity Parameters and Physical Properties of Organic Compounds from Molecular Structure Using SPARC*; EPA/600/R-03/030; Athens, GA, 2003; p 158.

3. Yalkowsky, S. H.; Dannenfelser, R. M. *The AQUASOL DATABASE of aqueous solubility, Version 5*; University of Arizona: Tucson, AZ, USA, 1992.

4. Lewis, K. M.; Archer, R. D., pKa values of estrone, 17-beta-estradiol and 2-methoxyestrone. *Steroids* **1979,** *34*, (5), 485-499.

5. Hansch, C.; Hoekman, D.; Leo, A.; Zhang, L. T.; Li, P., The expanding role of quantitative structure-activity relationships (QSAR) in toxicology. *Toxicol. Lett.* **1995,** *79*, (1-3), 45-53.

6. Lee, L. S.; Strock, T. J.; Sarmah, A. K.; Rao, P. S. C., Sorption and dissipation of testosterone, estrogens, and their primary transformation products in soils and sediment. *Environmental Science & Technology* **2003,** *37*, (18), 4098-4105.

7. Casey, F. X. M.; Shappell, N. W.; Hakk, H., Halogenated 17β-estradiol surrogates: Synthesis, estrogenic activity, and initial investigations of fate in soil/water systems. *Journal of Environmental Quality* **2017,** *46*, (4), 802-810.

8. Khan, E.; Subramania-Pillai, S., Interferences contributed by leaching from filters on measurements of collective organic constituents. *Water Research* **2007,** *41*, (9), 1841-1850.

9. Hammes, F.; Goldschmidt, F.; Vital, M.; Wang, Y.; Egli, T., Measurement and interpretation of microbial adenosine tri-phosphate (ATP) in aquatic environments. *Water Research* **2010,** *44*, (13), 3915-3923.

10. Milstead, R. P.; Nance, K. T.; Tarnas, K. S.; Egelhofer, K. E.; Griffith, D. R., Photochemical degradation of halogenated estrogens under natural solar irradiance. *Environmental Science: Processes and Impacts* **2018,** *20*, (10), 1350-1360.

11. Haag, W. R.; Hoigne, J., Singlet oxygen in surface waters. 3. Photochemical formation and steady-state concentrations in various types of waters. *Environmental Science & Technology* **1986,** *20*, (4), 341-348.

12. Schwarzenbach, R. P.; Gschwend, P. M.; Imboden, D. M., *Environmental Organic Chemistry*. 2nd ed.; John Wiley & Sons, Inc.: Hoboken, NJ, 2003.

13. Min, X.; Li, W.; Wei, Z.; Spinney, R.; Dionysiou, D. D.; Seo, Y.; Tang, C. J.; Li, Q.; Xiao, R., Sorption and biodegradation of pharmaceuticals in aerobic activated sludge system: A combined experimental and theoretical mechanistic study. *Chemical Engineering Journal* **2018,** *342*, 211-219.

14. Pomiès, M.; Choubert, J. M.; Wisniewski, C.; Coquery, M., Modelling of micropollutant removal in biological wastewater treatments: A review. *Science of the Total Environment* **2013,** *443*, 733-748.

15. Bjerg, P. L.; Brun, A.; Nielsen, P. H.; Christensen, T. H., Application of a model accounting for kinetic sorption and degradation to in situ microcosm observations on the fate of aromatic hydrocarbons in an aerobic aquifer. *Water Resources Research* **1996,** *32*, (6), 1831-1841.

16. Helbling, D. E.; Johnson, D. R.; Honti, M.; Fenner, K., Micropollutant biotransformation kinetics associate with WWTP process parameters and microbial community characteristics. *Environmental Science and Technology* **2012,** *46*, (19), 10579-10588.

17. Das, B. S.; Lee, L. S.; Rao, P. S. C.; Hultgren, R. P., Sorption and Degradation of Steroid Hormones in Soils during Transport: Column Studies and Model Evaluation. *Environmental Science and Technology* **2004,** *38*, (5), 1460-1470.

18. Hashimoto, T.; Murakami, T., Removal and degradation characteristics of natural and synthetic estrogens by activated sludge in batch experiments. *Water Research* **2009,** *43*, (3), 573-582.

19. Mashtare, M. L.; Green, D. A.; Lee, L. S., Biotransformation of 17α- and 17β-estradiol in aerobic soils. *Chemosphere* **2013,** *90*, (2), 647-652.

20. Xuan, R.; Blassengale, A. A.; Wang, Q., Degradation of estrogenic hormones in a silt loam soil. *Journal of Agricultural and Food Chemistry* **2008,** *56*, (19), 9152-9158.

21. Sobol, W. T., Analysis of variance for 'component stripping' decomposition of multiexponential curves. *Computer Methods and Programs in Biomedicine* **1993,** *39*, (3-4), 243-257.

22. Brenzonik, P. L., *Chemical Kinetics and Process Dynamics in Aquatic Systems*. Lewis: Boca Raton, 1994.

23. Lu, Y.; Chakrabarti, C. L.; Back, M. H.; Grégoire, D. C.; Schroeder, W. H.; Szabo, A. G.; Bramall, L., Methods for kinetic analysis of simultaneous, first-order reactions in waters: The kinetic model and methods for data analysis. *Analytica Chimica Acta* **1994,** *288*, (3), 131-139.

24. Plazinski, W.; Dziuba, J.; Rudzinski, W., Modeling of sorption kinetics: The pseudo-second order equation and the sorbate intraparticle diffusivity. *Adsorption* **2013,** *19*, (5), 1055-1064.

25. Kajjumba, G. W.; Emik, S.; Öngen, A.; Özcan, H. K.; Aydın, S., Modelling of Adsorption Kinetic Processes—Errors, Theory and Application. In *Advanced Sorption Process Applications*, Edebali, S., Ed. IntechOpen: Rijeka, 2018; p Ch. 10.

26. Martínez-Hernández, V.; Meffe, R.; Herrera López, S.; de Bustamante, I., The role of sorption and biodegradation in the removal of acetaminophen, carbamazepine, caffeine, naproxen and sulfamethoxazole during soil contact: A kinetics study. *Science of the Total Environment* **2016,** *559*, 232-241.
